# Dachsous-Fat signaling shapes the *Drosophila* wing through mechanical forces

**DOI:** 10.1371/journal.pbio.3003883

**Published:** 2026-07-14

**Authors:** Bipin Kumar Tripathi, Zhenru Zhou, Kenneth D. Irvine

**Affiliations:** Waksman Institute and Department of Molecular Biology and Biochemistry, Rutgers University, Piscataway, New Jersey, United States of America; The Francis Crick Institute, UNITED KINGDOM OF GREAT BRITAIN AND NORTHERN IRELAND

## Abstract

Proper organ shape is critical for function. The *Drosophila* wing normally adopts an elongated shape, but mutations in the Dachsous-Fat pathway result in rounder wings. The mechanism by which this occurs has remained unclear. Here, we show that Ds-Fat signaling shapes the wing during the larval stage, rather than during pupal development when morphogenetic rearrangements transform the developing wing disc into the adult wing. We further find that Ds-Fat alters tissue-wide stresses in the wing disc, and genetic manipulations that reduce cytoskeletal tension result in rounder wings, whereas increasing cytoskeletal tension produces more elongated wings. Reduced tension is also associated with less oriented growth during development. Notably, increased cytoskeletal tension partially rescues the rounder shape caused by *ds* knockdown. These results reveal a previously unrecognized mechanism by which Ds-Fat signaling determines wing shape, involving regulation of tissue tension to orient growth and shape the wing primordia during larval development.

## Abstract 2

The elongated shape of the Drosophila wing is regulated by the Dachsous-Fat signalling pathway, but the underlying mechanisms remain unclear. This study shows that mechanical forces regulated by this pathway modulate tissue-wide cytoskeletal tension to orient growth during larval development.

## Introduction

Forming organs of the correct size and shape is crucial for their normal function, and congenital disorders in which organs do not form correctly have a substantial impact on human health. In many cases, genes that are associated with congenital malformations play conserved roles in animal development (reviewed in [1,2]). Genes in the Dachsous-Fat pathway were first identified for their effects on the size and shape of wings and legs in *Drosophila* [3–8]. Mutations in the human homologs of *Drosophila* Fat and Dachsous (Ds), FAT4 and DCHS1, can result in congenital diseases associated with organ malformation, including Van Maldergem syndrome, Hennekam syndrome, and Mitral Valve Prolapse [9–11]. Gene-targeted mutations in murine Fat4 or Dchs1 genes similarly result in defects in formation of multiple organs [12–16].

Ds and Fat are large cadherin family proteins that initiate intercellular signaling to control organ growth, shape and planar cell polarity (PCP) [17–19]. Ds and Fat bind each other through their extracellular domains, with this binding modulated by Four-jointed (Fj)-mediated phosphorylation of cadherin domains [20–23]. Ds, Fj, and in some cases, Fat, are expressed in gradients across tissues, and their differential expression and binding interactions leads to polarized membrane localization of Ds and Fat [4,20,24–30]. Polarization of Ds and Fat leads to polarized membrane localization of a key downstream effector, Dachs [5,28,29,31]. Dachs is an unconventional myosin protein that mediates connections of Ds-Fat to regulation of Hippo signaling and PCP [5,27,28,32–36].

Ds-Fat signaling plays key roles in many different organs, but has been most intensively studied in the developing *Drosophila* wing. The wing develops from the wing imaginal disc, a cluster of ~30 cells specified during embryogenesis that undergo extensive proliferation, growing ~1,000 fold during larval development [37]. During pupal development, the wing disc undergoes morphogenetic changes including eversion, flattening, and expansion of the future wing blade to form the adult wing, wing hinge, and notum. The wing normally has an elongated, elliptical shape. This shape reflects both how the wing primordia, referred to as the wing pouch, grows within the developing larval wing disc, as well as morphogenetic processes that occur during pupal development. One key event within the pupa is a contraction of the wing hinge, which generates an anisotropic stress that pulls on the future wing blade and contributes to its elongation [38,39]. Loss of *ds*, *fat*, or other core components of Ds-Fat signaling results in wings that are rounder than wild-type wings. A potential explanation for this was suggested by examination of growth and spindle orientation during larval development. Growth orientation can be visualized by making marked clones of cells, which tend to be oriented along the proximal-distal axis of the developing wing; this orientation correlates with a preferential orientation of mitotic spindles along the proximal-distal axis. This orientation of mitotic spindles, and of clone growth, is lost in *ds*, *fat* or *dachs* mutants, which led to the suggestion that Ds-Fat signaling controls growth orientation, and ultimately wing shape, through effects on spindle orientation [40,41].

The suggestion that Ds-Fat signaling control wing shape through effects on spindle orientation was called into question by the discovery that spindle orientations are randomized by mutation of *mud*, but clone growth and wing shape are nonetheless normal in *mud* mutants [42]. How then might the effect of Ds-Fat on wing shape be explained? One clue comes from investigations of cellular contributions to tissue shear. As the wing disc grows during mid-third instar, three cellular behaviors account for most shear along the proximal–distal axis: oriented cell divisions, oriented cell rearrangements, and oriented cell shape changes. Intriguingly, cellular analysis of live discs cultured ex vivo revealed that the relative contributions of these processes can vary between individual discs [43]. Moreover, in *mud* mutant discs, the loss of oriented cell divisions was at least partially compensated for by an increased contribution of cell rearrangements to tissue shear [42]. These observations suggest that the cellular behaviors observed could be interchangeable responses to tissue stress.

Here, we revisit the question of how Ds-Fat signaling influences wing shape. We confirm that Ds-Fat signaling acts principally during larval stages to control adult wing shape. Our investigations reveal that *ds* and *ft* mutants alter the shape of the wing pouch from the earliest stages of its formation. This initial alteration of wing pouch shape is shared by mutations that disrupt Hippo signaling, but alterations in Hippo signaling, or in canonical PCP, cannot account for the influence of Ds-Fat on wing shape. Instead, we find that loss of Ds or Fat alters tissue stresses within the wing pouch, and that these alterations are associated with changes in the distribution of non-muscle myosin II. We further show that direct alterations of cytoskeletal tension can alter wing shape and can modulate the consequences of *ds* mutations on wing shape. Altogether, our observations support a hypothesis in which Ds-Fat signaling controls wing shape by patterning tissue stresses that shape the orientation of growth throughout larval development.

## Results

### Dachsous and Fat are required during larval development for wing shape

**Fig 1:**
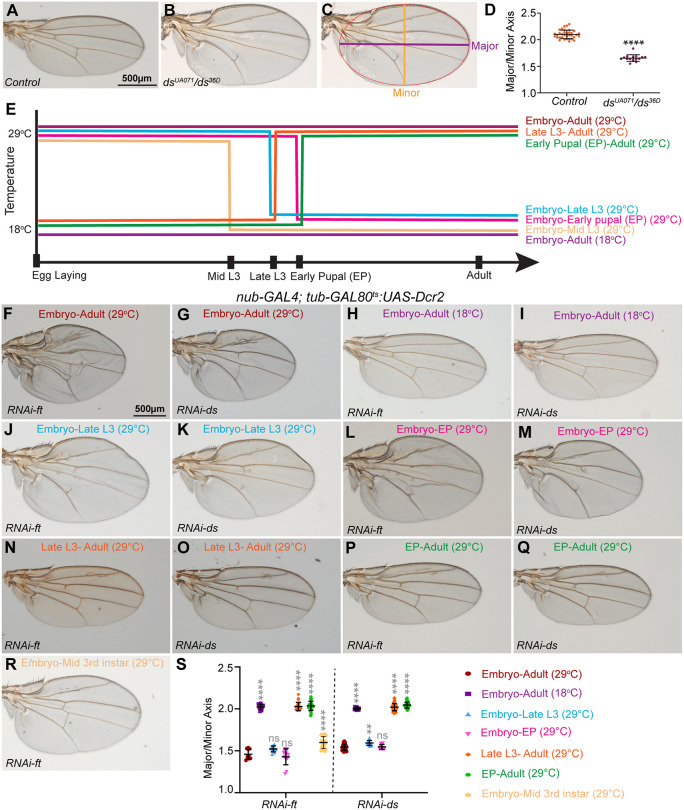
Effect of temporal knockdown of *fat* and *ds* on wing shape. **(A-C)** Male wings from *w*^*1118*^ **(A)**, *ds*^*UA071*^*/ds*^*36D*^ **(B,C)**. In C, the fitted ellipse (red) used to measure the major axis (purple line) and minor axis (orange line) is overlayed. Scale bar = 500 µm. **(D)** Histogram illustrating the Major/Minor axis ratio of control (*w*^*1118*^, *n* = 35) and *ds*^*UA071*^*/ds*^*36D*^ (*n* = 15). Error bar indicates mean ± s.d., the significance of differences by *t* test is indicated by black asterisks. **(E)** Schematic for temperature shift experiment to knockdown Fat (Ft) and Ds using the *GAL4/GAL80*^*ts*^ system at different developmental stages. Wandering third instar larvae (~8 to 12 h before puparium formation) were used as Late L3, and 0–8 h APF (starting from white pre-pupae) was used as early pupal stage (EP), 24 h before puparium formation at 18 °C was used as Mid L3. **(F–R)** Male wings from knockdown of Ft from embryo to adult (F; *n* = 10), Ds from embryo to adult (G; *n* = 28), Ft control (H; *n* = 11), Ds control (I; *n* = 10), Ft from embryo to late third instar (J; *n* = 11), Ds from embryo to late third instar (K; *n* = 11), Ft from embryo to early pupal (L; *n* = 13), Ds from embryo to early pupal (M; *n* = 11), Ft from Late L3 to adult (N; *n* = 22), Ds from Late L3 to adult (O; *n* = 50) Ft from early pupal to adult (P; *n* = 10), Ds from early pupal to adult (Q; *n* = 12), Ft from embryo to mid-L3 (R; *n* = 21). Scale bar = 500 µm. **(S)** Histogram quantifying wing shapes for wings as shown in F–O. Error bar indicates mean ± s.d, and the significance of differences relative to the Embryo-Adult (29 °C) condition calculated using one-way ANOVA on measurements from the number of wings indicated above is indicated in gray for *UAS-*RNAi-*ft* (left) and *UAS-*RNAi-*ds* (right). The data underlying the results presented in D and S are available in S1 Data.

Mutations in several components of the Ds-Fat pathway, including *ds*, result in rounder wings [4,5,30,40,41,44] (Fig 1A and 1B). This can be quantified by comparing the length of the wing to its width. To simplify these measurements, we fit a tracing of the outline of the wing to an ellipse and plotted the ratio of the major axis (length) of the ellipse to its minor axis (width) (Fig 1C). Using this approach, we measured a major/minor axis ratio of 2.1 for wild-type wings, and 1.6 for *ds* mutant wings (Fig 1D). Ds and Fat influence growth orientation during larval development [40,41], but also influence oriented cell behaviors during pupal development [45–47]. To distinguish larval versus pupal contributions of *ds* and *fat* to adult wing shape, we used conditional expression of RNAi transgenes. *UAS-RNAi*-*fat* or *UAS-RNAi*-*ds* transgenes were expressed under *nub-Gal4* control, which begins to be expressed in the developing wing blade and distal hinge during the second larval instar [48]. Temporal control of expression was provided using a ubiquitously-expressed temperature-sensitive GAL80 (tub-GAL80^ts^) to antagonize GAL4 [49]. When Fat or Ds were knocked down throughout most of larval development by keeping flies continuously at 29 °C (inactivating GAL80^ts^), then rounder adult wings were generated, similar to those observed in *ds* mutants (Fig 1E–1G and 1S). When *ds* or *fat* RNAi was suppressed by keeping flies continuously at 18 °C, then wing shapes similar to those in wild-type controls were observed (Fig 1H, 1I, and 1S). Knockdown of Fat or Ds from embryonic through late third instar larval stages (wandering third instar larvae, ~ 8–12 hours (h) before puparium formation) or early pupal stages (0–8 h after puparium formation) (Fig 1J–1M and 1S) resulted in rounder wings, like in *ds* mutants. Conversely, knockdown of Fat or Ds starting from late third instar larval or early pupal stages to adulthood resulted in wing shapes similar to wild-type controls (Fig 1N–1Q and 1S). These observations suggest that Ds and Ft are required during larval development, but not during pupal development, for normal wing shape.

To further evaluate the conclusion that wing shape is specified during larval development, we examined the recovery of protein expression after shifts from 29 to 18 °C. Due to limitations of antibody availability, these experiments were only performed for Fat. We found that Fat staining was recovered by 24 h after shifting to 18 °C, but not after only a 6 h shift (S1A–S1C Fig). As this indicates that Fat or Ds protein might not be fully recovered by the beginning of pupal development in our temperature downshift experiments, we also performed an additional downshift experiment in which larvae were shifted from 29 to 18 °C at 24 h before puparium formation. This resulted in adult wings that were almost, but quite, as round as those from animals with continuous knockdown of *fat* (Major/Minor axis of 1.6, versus 1.5 for continuous RNAi, Fig 1R and 1S).

### Ds-Fat signaling regulates the shape of the wing pouch in larval wing discs

**Fig 2:**
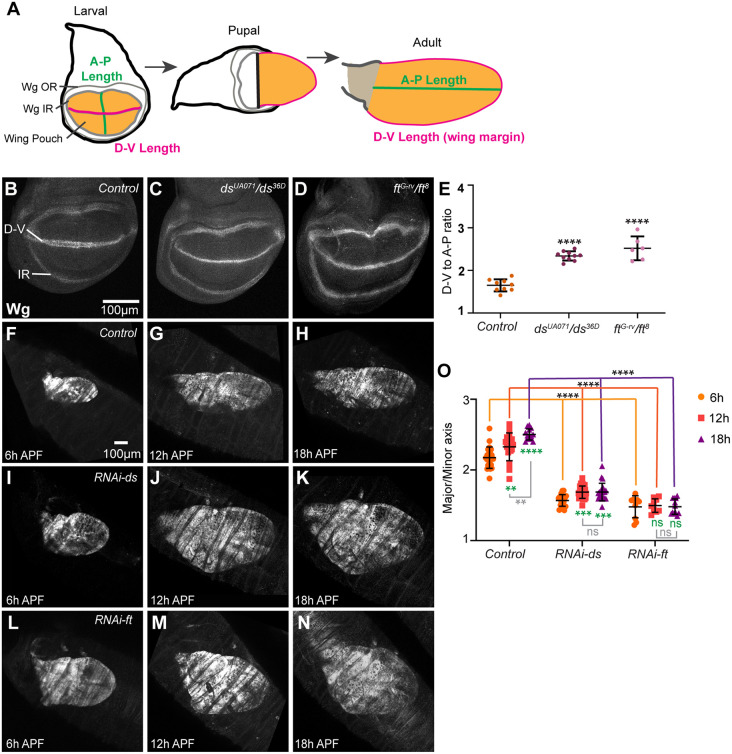
Altered wing pouch shape in *ds* and *fat* mutants. **(A**) Schematic showing relationship between wing disc and adult wing, with approximate locations of wing pouch (orange), D–V length (magenta) and A–P length (green), Wg outer (OR) and inner (IR) rings indicated. The D–V length in the wing pouch corresponds to the circumference of the adult wing (the wing margin), while the A–P length in the wing disc corresponds to twice the length of the adult wing, as the wing pouch folds in half along the D–V boundary. **(B–D)** Late third instar wing discs stained for Wg from *w*^*1118*^ (B; control, *n* = 9), *ds*^*UA071*^*/ds*^*36D*^ (C; *n* = 10), and *ft*^*G-rv*^*/ft*^*8*^ (D; *n* = 6). Scale bar = 100 µm. **(E)** Histogram illustrating the wing pouch shape for wing discs of genotypes shown in B–D. Error bar indicates mean ± s.d., the significance of differences relative to *w*^*1118*^, calculated using one-way ANOVA on measurements from the number of wing discs indicated above is indicated by black asterisks. **(F–N)** Pupal wings from *nub-GAL4:UAS-Dcr2/+; UAS-mCD8:RFP/+* (Control, F–H), *nub-GAL4:UAS-Dcr2/ UAS-RNAi-ds; UAS-mCD8:RFP/+* (I–K) or *nub-GAL4:UAS-Dcr2/ UAS-RNAi-ft; UAS-mCD8:RFP/+* (L–N) at 6 h (F, *n* = 23, I, *n* = 19, L, *n* = 10), 12 h (G, *n* = 17, J, *n* = 25, M, *n* = 9), and 18 h (H, *n* = 14, K, *n* = 22, N, *n* = 12) APF. Scale bar = 100 µm. **(O)** Histograms showing measurements of pupal wing shape. Error bar indicates mean ± s.d., the significance of differences to control at the same developmental stage is indicated by black asterisks, calculated by using one-way ANOVA on measurements from the number of wings indicated above, the significance of differences at later times relative to 6 h APF is indicated by green asterisks or ns (not significant), and between 12 and 18 h APF in gray. The data underlying the results presented in E and O are available in S1 Data.

The adult wing blade develops from a central region of the ventral half of the larval wing imaginal disc referred to as the wing pouch, which is demarcated by folds that form in the future wing hinge [37]. During metamorphosis, the wing pouch everts and folds in half, such that the dorsal–ventral (D–V) midline of the wing pouch (the D–V compartment boundary) becomes the margin (edge) of the adult wing (Fig 2A). To investigate whether the altered shape of the adult wing is reflected in the shape of the wing pouch, we compared wing pouch shape in wild-type versus *ds* or *fat* mutant wing discs. We used expression of Wingless (Wg) to analyze wing pouch shape, as it is expressed in two rings of cells in the developing hinge as well as in a line of cells along the D–V compartment boundary (Fig 2B). In adult wings, the location of the inner ring of Wg expression [50] corresponds roughly to the proximal edge of the region we measured (Fig 1C). To compare wing pouch shapes, we measured the D–V/A–P ratio, with the D–V length defined as the length of Wg expression along the D–V boundary within the inner ring of Wg expression (Fig 2A), and the A–P length defined as the width of the inner ring at its center (roughly along the A–P compartment boundary) from top (dorsal) to bottom (ventral). In wild-type wing discs at late third instar, the D–V/A–P ratio is ~1.7 (Fig 2B and 2E). In contrast, in *ds* mutant wing discs, the D–V/A–P ratio is ~2.4 and in *ft* mutant wing discs the D–V/A–P ratio is ~ 2.5 (Fig 2C–2E). As the D–V length in the wing disc corresponds to the circumference of the adult wing, while the A–P length is proportional to the proximal-distal length of the adult wing (Fig 2A), a larger D–V/A–P ratio in the wing disc corresponds to a rounder adult wing.

### Influence of Ds-Fat signaling on pupal wing shape

To further examine the inference that altered larval wing pouch shape accounts for altered adult wing shape, we examined developing pupal wings from wild-type, *ds* RNAi, and *fat* RNAi knockdown animals. For these experiments, we used nub-GAL4 driving the expression of UAS-mCD8:RFP to label developing pupal wings. To minimize disturbance of pupal wing morphology, developing wings were imaged live directly through the pupal case. We found that at the beginning of pupal development (white prepupae) the three-dimensional shape of the everting wing made it difficult to reliably measure wing shape. However, by 6 h after puparium formation (APF), the wing has everted and folded into opposing dorsal and ventral surfaces, and even at 6 h APF, *ds* and *fat* RNAi wings were substantially wider than control wings (Fig 2F, 2I, 2L, and 2O). The difference in shape was maintained as the wings flatten and expand (Fig 2G, 2H, 2J, 2K, and 2M–2O). The early change in pupal wing shape is consistent with the conclusion that *ds* and *fat* act during larval stages to regulate wing shape. We note that *ds* and *fat* RNAi wings appear not to elongate as much from 6 to 18 h APF (at 29 °C) as control wings, as the major/minor axis ratio increases in control wings more than in *ds* or *fat* RNAi wings (Fig 2F–2O). This might suggest roles for *ds* and *fat* in contributing to wing elongation during pupal development. However, given the genetic evidence that Ds-Fat signaling influences wing shape during larval growth, we favor the interpretation that the differences in pupal wing elongation from 6 to 18 h APF stem from consequences of Ds-Fat action during larval stages. For example, the overgrowth of the wing, which is particularly pronounced in the proximal wing and hinge in *ds* and *fat* mutants [27], might result in relatively less shear of the wing blade in response to the hinge contraction that contributes to pupal wing elongation [46].

### The influence of Ds-Fat signaling on wing pouch shape is visible at early third instar

**Fig 3:**
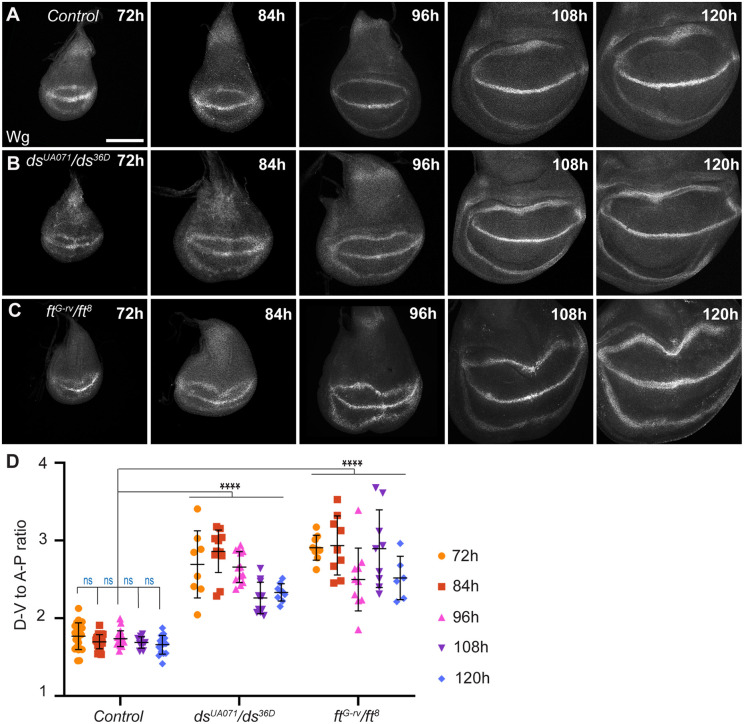
*ds* and *fat* mutants have altered wing pouch shapes throughout third instar. **(A–C)** Wing discs stained for Wg from **(A)** *w*^*1118*^, **(B)** *ds*^*UA071*^*/ds*^*36D*^, **(C)** *ft*^*G-rv*^*/ft*^*8*^ at 72 h (*n* = 23 *w*, 8 *ds*, 9 *ft*), 84 h (*n* = 22 *w*, 13 *ds*, 9 *ft*), 96 h (*n* = 20 *w*, 13 *ds*, 10 *ft*), 108 h (*n* = 10 *w*, 11 *ds*, 9 *ft*), and 120 h (*n* = 15 *w*, 9 *ds*, 6 *ft*) AEL. Scale bar = 100 µm. **(D)** Graph of wing pouch shape for discs described in A–C. Error bar indicates mean ± s.d., and significance of differences for different developmental stages of *w*^*1118*^ is relative to its 72 h stage and indicated as ns in blue; the significance of differences relative to *w*^*1118*^ at same developmental stage is indicated by black asterisks and were calculated using one-way ANOVA on measurements from the number of samples indicated above. The data underlying the results presented in D is available in S1 Data.

Ds-Fat signaling can influence the organization of growth during larval development, as the shapes of clones growing in *ds*, *fat* or *dachs* mutant wings are more rounded than clones growing in wild-type wings [40,41]. If the altered shape of the larval wing disc were solely a consequence of altered patterns of growth, then we reasoned that this altered shape would arise gradually over third instar, in conjunction with disc growth. To investigate this possibility, we examined the developmental profile of wing pouch shape throughout the third larval instar (at 72, 84, 96, 108, and 120 h after egg laying [AEL]), using analysis of the D–V/A–P ratio in wing discs stained for Wg expression. In wild-type wing discs, the D–V/A–P ratio was relatively consistent from the earliest time that a ring of Wg expression in the hinge could be clearly identified [37] (1.8 at early third instar, 72 h AEL) through the end of the third larval instar (1.7 at 120 h AEL, Fig 3A and 3D). Contrary to our expectations, in *ds* or *fat* mutants the D–V/A–P ratio was already abnormal at 72 h AEL, at 2.6 for *ds* and 2.9 for *fat* (Fig 3B–3D). The D–V/A–P ratio in *ds* or *fat* mutants reduced slightly as the discs grew, but remained significantly higher than that in wild-type controls throughout third instar. These observations indicate that Ds-Fat signaling influences wing shape during early development of the wing pouch, in addition to its effects during wing growth.

Our measures of the D–V/A–P ratio in wing discs are approximations based on projections through a tissue that begins relatively flat but becomes curved and folded at late third instar [37] (S2 Fig). To confirm that our assessment of the relative differences in D–V/A–P ratio are not skewed by this curvature and folding, we also measured distances in three dimensions across curved surfaces (3D) at 72 and 120 h AEL in both *wild-type* and *ds* mutant wing discs (S2 Fig). Measured in 3D, the D–V/A–P ratio at 72 h was 1.8 in control discs and 2.8 in *ds* mutant discs, whereas at 120 h it was 0.9 in control discs and 1.3 in *ds* mutant discs. These absolute values measured in 3D differ from the 2D measures largely because of the folds surrounding the wing pouch at 120 h (S2 Fig). Nonetheless *ds* mutant discs still have a larger D–V/A–P ratio than control discs at both 72 and 120 h. Moreover, the fold difference determined is similar regardless of the measurement method (i.e., at 72 h *ds* mutants are 2.6/1.8 = 144% wider in 2D measures and 2.8/1.8 = 155% wider in 3D measures, and at 120 h *ds* mutants are 2.6/1.7 = 153% wider in 2D measures and 1.3/0.9 = 144% wider in 3D measures). Thus, the 2D measures provide a simple method for qualitative determinations of relative wing pouch shape.

### The influence of Ds-Fat on wing shape is not accounted for by Hippo signaling

Ds-Fat signaling regulates distinct downstream processes including Hippo signaling and PCP. To investigate whether effects on Hippo signaling contribute to the influence of Ds-Fat on wing and wing disc shape, we examined flies mutant for the Hippo pathway regulator *expanded (ex)*, as adult flies can be recovered from animals mutant for *ex*^*e1*^, an amorphic allele of *ex* [51]. *ex*^*e1*^ adult wings are rounder than wild-type wings, with a major/minor axis ratio of 1.8 (S3A, S3C, and S3I Fig), but not as round as *ds* or *fat* mutant or RNAi wings (Fig 1B and 1D). Null mutations of *warts* (*wts*) and *hippo* (*hpo*) are lethal, but wings from animals in which *wts* or *hpo* were knocked down during wing development by RNAi expression under nub-Gal4 control were similarly intermediate in shape between wild-type controls and *ds* or *fat* mutants or RNAi (S3B, S3D, and S3I Fig), with a major/minor axis ratio of 1.8 and 1.9 respectively. The observations that *ds* mutants, *ex* mutants, *fat* RNAi, *wts* RNAi or *hpo* RNAi have similar effects on wing size (S3L Fig), but distinct effects on wing shape, suggest that Hippo signaling contributes to, but does not fully explain, the influence of *ds* or *fat* on adult wing shape.

We also examined the consequences of impairing Hippo signaling on wing disc shape. In *ex*^*e1*^ mutants, or in animals mutant for *wts* (*wts*^*P2*^*/ wts*^*X1*^, a hypomorphic combination of *wts* alleles that survives until pupal stages) we observed that the initial wing disc shape at early third instar is elongated along the D–V boundary (S3E, S3F, and S3J Fig), as observed in *ds* or *fat* mutants (Fig 3B and 3C). This suggests that the initial elongation of the wing pouch in *ds* or *fat* mutants could be due to impairment of Hippo signaling. However, as the wing disc grows, the D–V/A–P ratio in *ex*^*e1*^ or *wts* mutants gradually declines, such that by the end of third instar it is similar to that in wild-type controls (S3E, S3F, and S3J Fig). We also examined wing disc shape in animals expressing *wts* RNAi or *hpo* RNAi under nub-Gal4 control. These were not significantly different in shape from control wings discs at early stages (72–96 h AEL) (S3G, S3H, and S3K Fig), presumably because nub-Gal4 driven expression is too limited or too weak at this time to result in the elongated shapes observed in *wts* or *ex* mutants. At older stages (108–120 h AEL) *wts* RNAi wing discs actually had a slightly lower D–V/A–P ratio (1.5) than control discs (1.7), whereas *hpo* RNAi disc shape was similar to that in controls (S3G, S3H, and S3K Fig). These results imply that during wing disc growth, Ds-Fat has effects on wing pouch shape that are distinct from its effects on Hippo signaling.

During pupal development, the hinge region of the developing wing contracts [46]. As the distal region of the wing is attached to apical extracellular matrix, this contraction contributes to elongation of the wing blade [38,39]. This led us to consider whether the increased size of the wing in *ex* mutants, *hpo* RNAi, or *wts* RNAi might be a factor in their rounder adult wing shape. To investigate this, we increased growth through a distinct mechanism, by expressing an activated form of the Insulin receptor (*InR*^*CA*^) under *nub-GAL4* control. Expression of *InR*^*CA*^ in the wing under *nub-Gal4* control results in larger adult wings, similar in size to those generated by *fat* knockdown (S4A–S4D Fig). However, instead of being rounder, adult wings expressing *InR*^*CA*^ were actually slightly more elongated than control wings (S4A, S4B, and S4E Fig). Wing discs expressing *InR*^*CA*^ were similar to controls in shape during early larval development (72–96 h AEL), but as the wing disc grows the D–V/A–P ratio in *UAS-InR*^*CA*^ *nub-GAL4* declined slightly, such that by the end of third instar the wing pouch is elongated along the A–P axis compared to controls (S4F and S4G Fig), which correlates with the observed elongation of adult wings (S4B and S4E Fig). Thus, increased roundness of the adult wing in Hippo pathway mutants is not simply a consequence of increased wing size.

### Over-expression of Ds or Fat also alter wing pouch shape

Over-expression of Fat or Ds, or mutation of the downstream effector *dachs*, is associated with formation of wings that are both smaller and rounder than wild-type wings (S5A–S5C and S5E Fig) [5,20,31]. To investigate whether the altered adult wing shape is reflected in altered wing pouch shape in the larval disc, we used *nub-Gal4* to drive expression of *UAS-fat* during wing development. Over-expression of Fat was associated with a higher D–V/A–P ratio, 1.9 even at 72 h AEL, and the D–V/A–P ratio increased further during wing disc growth (2.1 at 120 h AEL) (S5F and S5H Fig). Over-expression of Fat is associated with removal of Dachs from cell membranes [5], and we also observed an elevated D–V/A–P ratio in *dachs* mutant wing discs throughout larval development (S5G and S5I Fig). The altered shape of the wing pouch in these genotypes is consistent with the rounder adult wings that they form. Notably, other genotypes that reduce Hippo signaling, such as downregulation of *jub* or *zyx*, or over-expression of *wts*, are associated with narrower adult wings rather than wider adult wings (S5D and S5E Fig) [52]. This supports the inference that the influence of *fat* or *dachs* on wing shape is not due to their impact on Hippo signaling.

### Ds regulates patterns of tissue stress

**Fig 4:**
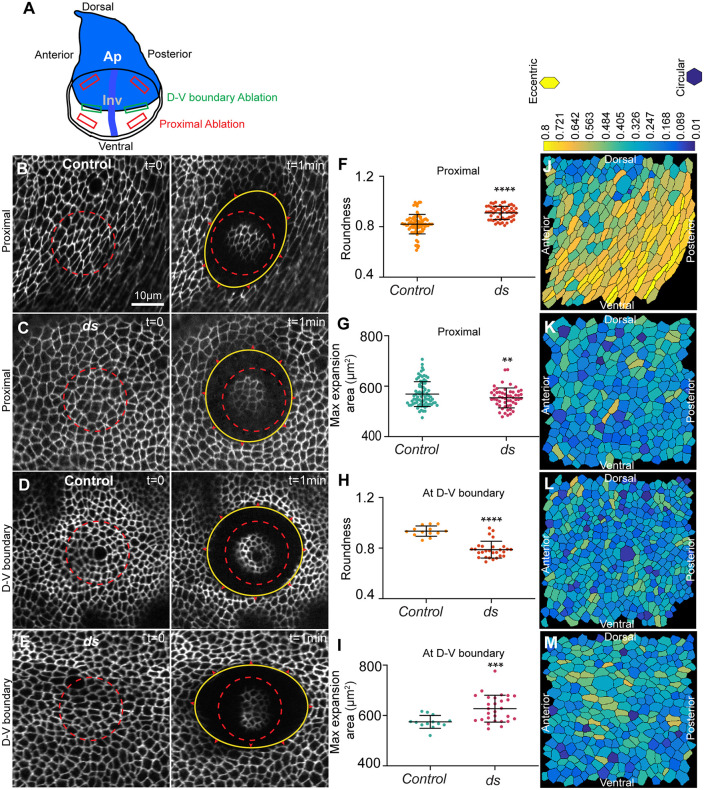
Mapping stress in *ds* mutants by circular ablation. **(A)** Schematic of late third instar wing disc, with expression patterns of Ap (blue) and Inv (dark blue), and approximate regions for circular ablation in the proximal wing pouch (red) and near the D–V boundary (green) indicated. **(B)** Wing disc expressing Ecad:GFP at 108 h from control tissue (*Ecad:GFP/ inv-BFP ap-BFP*) showing the tissue before (*t* = 0) and after (t = 1 min) a proximal circular ablation (*n* = 83). **(C)** Wing disc expressing Ecad:GFP at 108 h from *ds* mutant (*ds*^*UA071*^*/ds*^*36D*^*;Ecad:GFP/ inv-BFP ap-BFP*) showing the tissue before and after a proximal circular ablation (*n* = 54). **(D)** Wing disc expressing Ecad:GFP at 108 h from *control* showing the tissue before and after a circular ablation at D–V boundary (*n* = 13). **(E)** Wing disc expressing Ecad:GFP at 108 h from *ds* mutant showing the tissue before and after a circular ablation at D–V boundary (*n* = 28). Scale bar = 10 µm. The initial circular ablation region is marked by dotted red circle; the outer edge of the cut after 1 min is marked by a solid yellow ellipse. **(F–I)** Histograms plotting shape (F, H) and size (G, I) of the cut regions at their maximal expansion. Error bars indicate mean ± s.d., the significance of differences relative to control tissue by *t*-tests on measurements from the number of samples indicated above is indicated by black asterisks. **(J–M)** Heatmaps showing cell eccentricity on segmented cells (from B to E), colored according to eccentricity using the scale at top. The data underlying the results presented in F, G, H and I are available in S1 Data.

The determination that randomizing spindle orientation does not alter wing shape [42], together with observations that variations in the contribution of spindle orientation to shear during wing disc growth can be compensated for by cell rearrangements or cell shape changes [42,43], suggest that the primary influence of *ds* and *fat* might be on stress patterns in the developing wing disc. To investigate whether stress patterns are altered in *ds* mutants, we used laser cutting of cell junctions [53,54]. We first made circular cuts with a 10 µm radius (encompassing ~ 25–30 cells) in wing discs at 108 h AEL. If the cut region is under tension, then the outer edge will expand as it is pulled by neighboring cells, with the extent of expansion correlating with the level of external tension and the shape revealing whether this tension is isotropic (yielding circular expansion) or anisotropic (yielding elliptical expansion). We made cuts in proximal or D–V boundary regions (Fig 4A) and measured the size and shape of the cut regions at their maximal expansion (which occurred ~1 min after cutting). In proximal regions of wild-type controls, cut expansion after circular ablation resulted in an enlarged elliptical shape, revealing anisotropic stress (Fig 4B, 4F, and 4G). Near the D–V boundary of wild-type controls, cut expansion after circular ablation also resulted in an enlarged elliptical shape, of similar size but less elliptical than in proximal regions (Figs 4D, 4H, 4I, S6A, and S6C). When we made cuts in proximal regions of *ds* mutant wing discs, cut expansion resulted in shapes that were smaller and rounder than those in wild type discs (Figs 4C, 4F, 4G, S6B, and S6D). Thus, in this region of *ds* mutants, tissue stress is lower and more isotropic. Conversely, near the D–V boundary, cut expansion after circular ablation in *ds* mutants resulted in enlarged areas that are larger and more elliptical than those in wild type discs (Figs 4E, 4H, 4I, S6B, and S6D). Thus, in this region of *ds* mutants, tissue stress is higher and more anisotropic. Stress anisotropy often correlates with cells shape, with anisotropic stress associated with more elongated cells. Consistent with this, quantification of cell eccentricity revealed that cells are less elongated in proximal regions in *ds* mutant wing discs as compared to wild-type cells in this region (Fig 4B, 4C, 4J, and 4K) [55,56]. To confirm that our inferences of relative tissue stress obtained from circular ablations correlate with differences in junctional tension, we also examined recoil velocity after laser cutting of individual cell junctions, which is proportional to junctional tension [54]. This analysis revealed that recoil velocity in *ds* mutants is lower than in control discs in proximal regions and higher than in control discs near the D–V boundary (S6E–S6J Fig), consistent with the inference from circular ablations.

### Influence of Ds-Fat signaling on myosin localization

**Fig 5:**
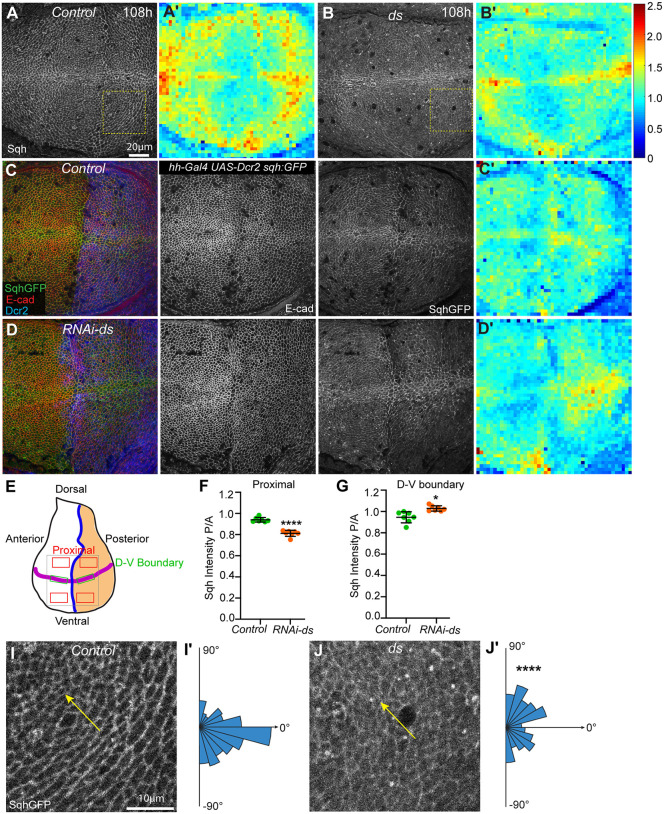
Altered myosin in *ds* mutants. **(A, B**) Wing discs expressing Sqh:GFP at 108 h from **(A)** control (Sqh:GFP/+) and **(B)** *ds*^*UA071*^*/ds*^*36D*^*; Sqh:GFP/+.* **(C, D)** Wing discs expressing Sqh:GFP and *hh-GAL4:UAS-Dcr2* in control **(C)**, and in *RNAi-ds* **(D)***.* Scale bar for A–D = 20 µm. Panels marked by prime symbols show heat maps of Sqh:GFP intensity normalized to E-cad. Scale for the heat map is at top right. **(E)** Schematic of wing disc showing approximate location of proximal (red boxes) and D–V boundary (green boxes) Sqh intensity measures for quantitation. **(F, G)** Graph of relative Sqh intensity in proximal or D–V boundary regions for control and RNAi *ds* discs as described in C,D, form locations indicated in E. Error bar indicates mean ± s.d., and significance of differences is indicated by asterisks. **(I, J)** Measures of Sqh junctional polarity in control (I) or *ds* mutant (J) wing discs. Images show examples of myosin localization (from the boxed regions in A, B), panels marked prime show graphs of polarity measures, where 0° = radial (along yellow arrow in images), from *N* = 235 cells (in 8 discs) for control and 208 cells (in 7 discs) for *ds*. The significance of difference in distributions was calculated using a Kolmogorov–Smirnov test. The data underlying the results presented in F, G, I′, and J′ are available in S1 Data.

The distribution of myosin is a key driver of, and generally correlates with, tension in the actin cytoskeleton. To determine whether the altered stress patterns we detected in *ds* mutants could be explained by changes in myosin distribution, we examined Sqh:GFP, a GFP-tagged myosin light chain (encoded in *Drosophila* by the *spaghetti squash* gene, *sqh*)*.* Levels of Sqh:GFP at cell-cell junctions were quantified and compared with levels of E-cadherin (E-cad). Compared to control wing discs at 108 h AEL, *ds* mutant discs appear to have relatively higher junctional myosin near the D–V boundary, and lower junctional myosin in the rest of the wing pouch (Fig 5A and 5B), consistent with our observations of higher tissue tension near the D–V boundary and lower tissue tension in proximal regions. To confirm these differences, we also examined myosin distribution in wing discs in which *ds* was knocked down in posterior cells under *hh-Gal4* control, leaving anterior cells as an internal control. Quantitation revealed relatively increased levels of junctional myosin near the D–V boundary and reduced levels of junctional myosin in proximal regions in the *ds* knockdown compartment (Fig 5C–5G).

In proximal regions of the wing pouch, Myosin levels on cell-cell junctions are polarized, with higher levels on radial junctions and lower levels on tangential junctions [55,56] (Fig 5I). In *ds* mutants, we observed that not only are myosin levels lower, it is also less polarized (Fig 5I and 5J). While this might suggest a direct role for Ds in polarizing myosin, observations that myosin remains polarized in the absence of the downstream effector Dachs make this less likely [53,56]. Instead, as it has been hypothesized that myosin polarization occurs in response to growth patterns that stretch cells [55,56], the loss of myosin polarity could be an indirect consequence of altered growth patterns and tissue stress in *ds* mutants.

### Changes in cytoskeletal tension alter wing shape and growth orientation

**Fig 6:**
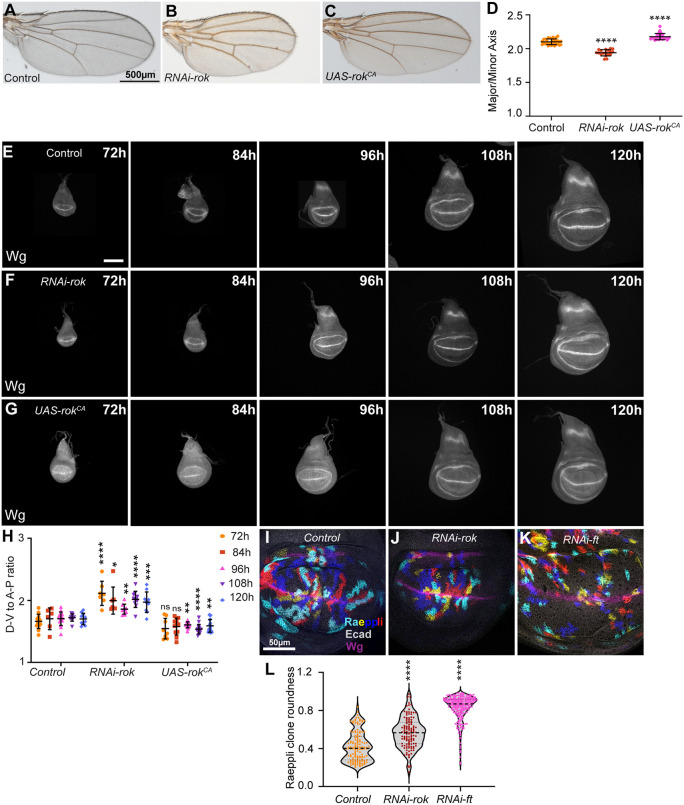
Influence of cytoskeletal tension on wing shape and growth orientation. **(A–C)** Male wings from *nub-GAL4:UAS-Dcr2/+* (A; *n* = 32), *nub-GAL4:UAS-Dcr2/ UAS-RNAi-rok* (B; *n* = 18), and *nub-GAL4:UAS-Dcr2/UAS-rok*^*CA*^ (C; *n* = 33). Scale bar = 500 µm. **(D)** Histogram quantifying adult wing shape for wings described in A–C. Error bar indicates mean ± s.d., the significance of differences relative to control, calculated by one-way ANOVA, is indicated by asterisks. **(E–G)** Wing discs stained for Wg from (E) *nub-GAL4:UAS-Dcr2/+* at 72 h AEL (*n* = 14), 84 h AEL (*n* = 6), 96 h AEL (*n* = 15), 108 h AEL (*n* = 11), and 120 h AEL (*n* = 10), (F) *nub-GAL4:UAS-Dcr2/ UAS-RNAi-rok* at 72 h AEL (*n* = 7), 84 h AEL (*n* = 7), 96 h AEL (*n* = 9), 108 h AEL (*n* = 11), and 120 h AEL (*n* = 10), (G) *nub-GAL4:UAS-Dcr2/UAS- rok*^*CA*^ at 72 h AEL (*n* = 8), 84 h AEL (*n* = 11), 96 h AEL (*n* = 15), 108 h AEL (*n* = 14) and 120 h AEL (*n* = 18 Scale bar = 100 µm. **(H)** Histogram quantifying wing pouch shape for wing discs described in E-G. Error bars indicate mean ± s.d., and the significance of differences relative to *nub-GAL4:UAS-Dcr2/+* (control) at the same developmental stage, calculated by t tests, is indicated by ns or black asterisks. **(I–K)** Third instar larval wing discs showing labeled clones using Raeppli technique from control (H, *n* = 13 discs), RNAi-rok (I, *n* = 14 discs) RNAi-fat (J, *n* = 19 discs). Scale bar = 50 µm. **(L)** Quantification of clone roundness from discs described in I–K, by violin plot, *N* = 132 (control), 122 (RNAi-rok), and 243 (RNAi-fat) clones. Error bar indicates mean ± s.d., the significance of differences relative to control, calculated by one-way ANOVA, is indicated by asterisks. The data underlying the results presented in D, H, and L are available in S1 Data.

Our examination of myosin distribution and tissue stress suggest that the Ds-Fat pathway alters wing shape by modulating tissue stress patterns. To further evaluate this hypothesis, we examined the consequences of genetically altering cytoskeletal tension on wing shape. We note that earlier studies suggest that altered tension can impact wing shape. For example, when Rho-associated kinase (Rok), which phosphorylates and activates myosin [57], is knocked down throughout the developing wing (UAS-RNAi-rok nub-Gal4) then wings appear both smaller and rounder [58]. We have extended these observations by measuring wing shapes and by analyzing the shape of the wing pouch during larval development. Quantitation confirmed that knockdown of *rok* results in rounder wings (Fig 6B and 6D). Conversely, increasing Rok activity by expressing an activated form of Rok comprising the catalytic domain (Rok.CA) resulted in flies with elongated wings (Fig 6C and 6D). To examine if these effects on adult wing shape are also reflected in larval wing pouch shape we measured the D–V/A–P ratio in wing discs from 72 to 120 h AEL. Decreasing Rok levels using RNAi-rok resulted in an increased D–V/A–P ratio, which was visible from early larval stages (72 h AEL) and remained elevated throughout larval development (Fig 6F and 6H). Conversely, increasing Rok activity in the wing discs resulted in a slightly decreased D–V/A–P ratio compared to controls, and the difference was statistically significant at later stages of development (Fig 6G and 6H).

During normal development, growth within the wing pouch is preferentially oriented in a proximal-distal direction, as revealed by the elongation of marked clones [40]. *ds* or *fat* mutants randomize the orientation of growth, resulting in rounder clones that are no longer oriented along the proximal-distal axis [40,41]. To evaluate the contribution of cytoskeletal tension to oriented growth, we examined clones of cells in *nub-Gal4 UAS-RNAi-rok* wing imaginal discs and compared them to clones in wild-type and in *nub-Gal4 UAS-RNAi-fat* wing discs. Clones were marked using the Raeppli technique, which generates clones labeled with one of four different fluorescent proteins [59]. Clones of cells in *rok* knockdown discs were rounder than those in wild-type discs, supporting a key role for cytoskeletal tension in orienting growth during wing development (Fig 6I, 6J, and 6L). However, they were not as round as those in *fat* RNAi discs (Fig 6K and 6L), consistent with the observation that *rok* RNAi wings are not as round as *fat* RNAi wings.

### Increased cytoskeletal tension modulates the shape of *ds* knock-down wings

**Fig 7:**
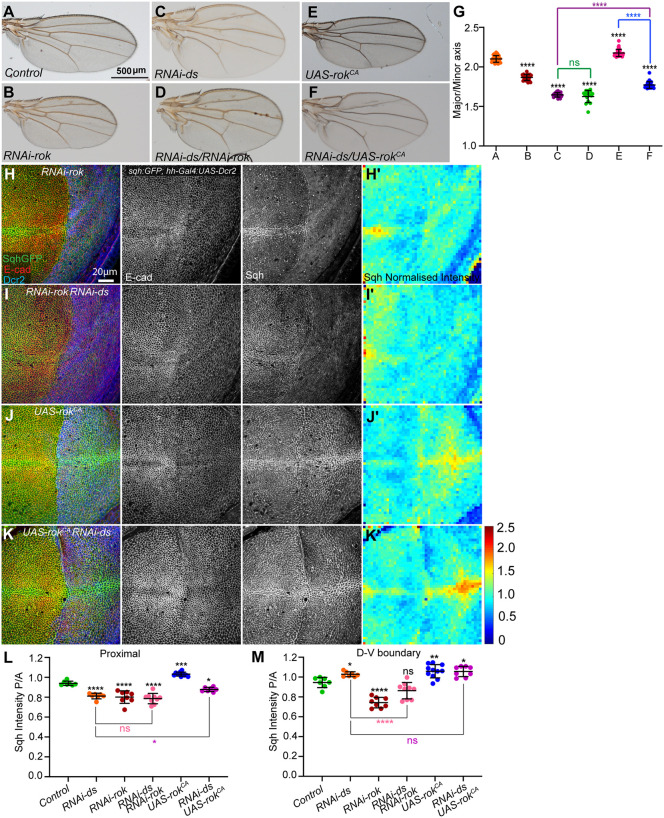
Increasing cytoskeletal tension partially rescues *ds* wing shape. **(A–F)** Male wings from *nub-GAL4:UAS-Dcr2/+* (control) (A; *n* = 32), *UAS-RNAi-rok* (B; *n* = 23), *UAS-RNAi-ds* (C; *n* = 19), *UAS-RNAi-rok UAS-RNAi-ds* (D; *n* = 17), *UAS-rok*^*CA*^ (E; *n* = 33), and *UAS-rok*^*CA*^ *UAS-RNAi-ds* (F; *n* = 27). Scale bar = 500 µm. **(G)** Histogram quantifying shape for adult wings described in A-F. Error bar indicates mean ± s.d., the significance of differences compared to control by one-way ANOVA is indicated by black asterisks and between *UAS-RNAi-rok* and *UAS-rok*^*CA*^ on *ds* knock-down is shown in green and blue, respectively and between *UAS-RNAi-ds* and *UAS-RNAi-ds* along with *UAS-rok*^*CA*^ in purple. **(H–K)** Wing discs expressing Sqh:GFP along with *hh-GAL4:UAS-Dcr2* in *UAS-RNAi-rok* **(H)**, in *UAS-RNAi-rok*, *UAS-RNAi-ds* **(I)**, in *UAS-rok.CA* **(J)**, and in *UAS-rok.CA*, *UAS-RNAi-ds* **(K)***.* Scale bar: 20 µm. Panels marked by prime symbols show heat maps of Sqh:GFP intensity normalized to E-cad. Scale for the heat map is at bottom right. **(L, M)** Graphs of relative Sqh intensity in proximal (L) or D–V boundary (M) regions for control and RNAi *ds* discs as described in Fig 5 together with discs as described in panels H–K, from locations as indicated in Fig 5E. Error bar indicates mean ± s.d., and significance of differences relative to control is indicated by black symbols, between *ds* RNAi and *ds* RNAi *rok* RNAi by pink symbols, and between *ds* RNAi and *ds* RNAi rok.CA by purple symbols. The data underlying the results presented in G, L and M are available in S1 Data.

Observations that loss of *ds* alters stress patterns in wing discs, together with observations that direct manipulation of cytoskeletal tension alters clone growth and wing shape, led us to investigate whether manipulating cytoskeletal tension could modify the consequences of *ds* knockdown on wing shape. We found that knocking down *rok* together with *ds* did not further increase wing roundness (Fig 7A–7D and 7G). Conversely, increasing Rok activity by expressing Rok.CA in *ds* knockdown wings partially rescued wing shape, as wings were elongated compared to *ds* knockdown wings (Fig 7C and 7E–7G). This implies that the reduced cytoskeletal tension in *ds* knockdown wings in proximal regions contributes to the effect of *ds* on wing shape.

We also examined myosin levels in posterior cells of wing discs in which altered cytoskeletal tension was combined with *ds* knock down*.* Knock down of *rok* in combination with *ds*-RNAi did not further reduce Sqh levels in proximal regions, but did reduce the elevated Sqh levels at the D–V boundary (Fig 7H, 7I, 7L, and 7M). Increasing cytoskeletal tension in combination with *ds*-RNAi by expressing Rok.CA significantly elevated the low Sqh levels in proximal regions, but not the elevated Sqh levels at the D–V boundary (Fig 7J–7M). That is, in *ds* RNAi wing discs, increased Rok reversed the low myosin in proximal regions but did not further increase the elevated myosin near the D–V boundary, whereas decreased Rok reversed the elevated myosin near the D–V boundary but did not further decrease the low myosin in proximal regions. Together with the analysis of wing shapes, these observations implicate the reduction in myosin levels and cytoskeletal tension in proximal regions as a contributor to altered wing shape in *ds* mutants.

### Dachs modulates myosin and interacts with altered Rok activity to shape wings

**Fig 8:**
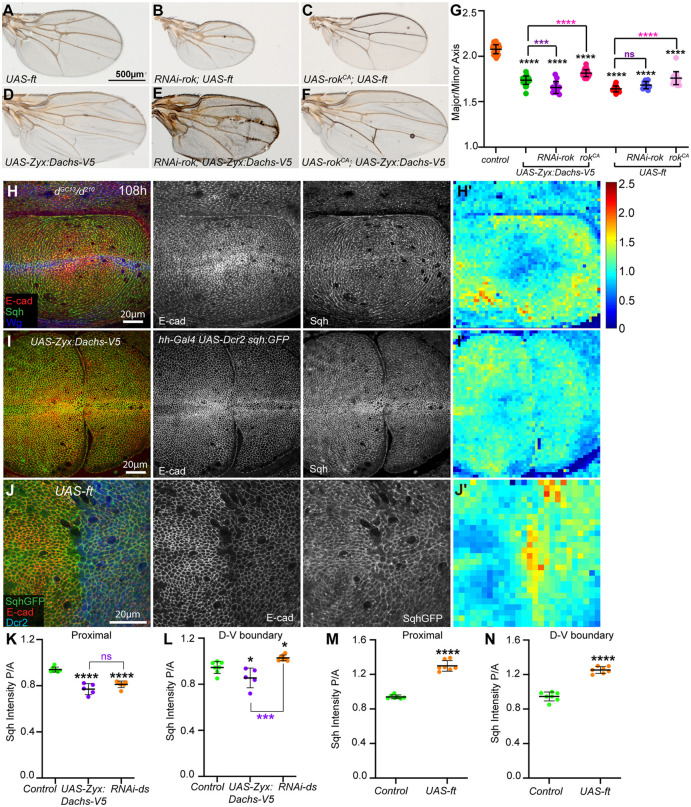
Influence of Dachs manipulation on myosin and interactions between them in modulating wing shape. **(A–F)** Male wings from *UAS-fat* (A; *n* = 21), *UAS-RNAi-rok*, *UAS-fat* (B; *n* = 11), *UAS-rok.CA*, *UAS-fat* (C; *n* = 16), *UAS-Zyx:Dachs-V5* (D; *n* = 8), *UAS-RNAi-rok*, *UAS-Zyx:Dachs-V5* (E; *n* = 11), and *UAS-rok.CA*, *UAS-Zyx:Dachs-V5* (F; *n* = 23). Scale bar = 500 µm. **(G)** Histogram quantifying shape for wings described in A–F. Error bar indicates mean ± s.d., the significance of differences relative to control, calculated by one-way ANOVA, is indicated by black asterisks, and differences between presence or absence of *UAS-RNAi-rok* and UAS-rok.CA is indicated by purple and pink symbols, respectively. **(H–J)** Wing discs expressing Sqh:GFP at 108 h from *d*^*210*^*/ d*^*GC13*^*; Sqh:GFP/+* (H), *hh-GAL4 UAS-Dcr2 UAS-Zyx:Dachs-V5* (I), *hh-GAL4 UAS-fat* (J). Panels marked by prime symbols show heat maps of Sqh:GFP intensity normalized to E-cad. Scale for the heat map is at top right. **(K–N)** Graphs of relative Sqh intensity in proximal (K,M) or D–V boundary (L,N) regions for control, UAS-Zyx:Dachs and RNAi *ds* discs (K,L) or UAS-ft discs (M,N) from locations as indicated in Fig 5E. Error bar indicates mean ± s.d., and significance of differences relative to control is indicated by black symbols, between UAS-Zyx:Dachs and *UAS-RNAi-d*s by purple symbols. The data underlying the results presented in G, K, L, M and N are available in S1 Data.

The myosin family protein Dachs plays in a key role in connecting Ds-Fat signaling to regulation of Hippo signaling and PCP. Dachs has also been reported to correlate with junctional tension [36,41], and to destabilize myosin in pupal wings [45]; these observations imply connections between Dachs and cytoskeletal tension, although not in a consistent direction. To investigate potential contributions of Dachs to regulation of myosin and wing shape, we examined myosin levels in *dachs* mutant wing discs, and in wing discs in which Dachs was constitutively recruited to cell junctions by fusion with Zyxin (Zyx) [60]. Expression of Zyx:Dachs in posterior cells was associated with a relative decrease in proximal myosin levels (Fig 8I and 8K), reminiscent of the reduced proximal myosin observed when *ds* is knocked down. *dachs* mutants appear to have an opposite phenotype, with myosin appearing lower in the center of the wing pouch as compared to more proximal regions (Fig 8H). As we found that available *dachs* RNAi lines were either not effective or had off-target effects, we instead used *UAS-ft* to remove Dachs protein from cell junctions. This did not visibly reduce relative myosin levels near the D–V boundary (Fig 8J and 8N), although the interpretation was complicated by a reduction of E-cad levels (Fig 8J, E-cad levels have previously been reported to increase when Fat is removed [61]).

When expressed in adult wings under *nub-Gal4* control, *UAS-fat* leads to smaller, rounder wings, similar to *dachs* mutants, whereas Zyx:Dachs expression leads to larger, rounder wings, reminiscent of *ds* or *fat* mutants [60,62] (Fig 8A, 8D, and 8G). Wings expressing Zyx:Dachs had a major/minor axis ratio of 1.7, significantly rounder than control wings (2.1) and slightly less round than *ds* mutant wings (1.6). Wings expressing Zyx:Dachs became slightly rounder when co-expressed with rok RNAi, and slightly less round when co-expressed with Rok.CA (Fig 8D–8G). Wings expressing UAS-fat had a major/minor axis ratio of 1.6, which was not further decreased by co-expression with rok RNAi, but which was partially reversed (to a major/minor axis ratio of 1.8) by co-expression with rok.CA (Fig 8A–8C and 8G).

### Altered cytoskeletal tension modulates shape in wings with decreased Hippo signaling

We also compared the impact of reduced cytoskeletal tension on wings from animals with impaired Hippo signaling by expressing *rok* RNAi in *ex* mutant or *hpo* knockdown flies. This further increased the roundness of the adult wing (S7A–S7D and S7F Fig). Moreover, decreasing Rok levels in *ex* mutants increased the D–V/A–P ratio in wing discs at later stages of larval development (S7E and S7G Fig), consistent with this increased roundness. We also examined myosin localization in *ex* mutant wing discs, and in wing discs with *hpo* RNAi knockdown in posterior cells. In *hpo* RNAi, myosin levels actually appear elevated in the proximal region (S7I and S7J Fig), opposite to the reduced myosin observed in *ds* mutants or with *ds* RNAi (Fig 5). The observations that Ds-Fat pathway mutants have distinct effects on myosin distribution as compared to Hippo pathway mutants suggests that distinct effects on tension, presumably mediated through Dachs, contribute to the increased roundness of Ds-Fat pathway mutants as compared to Hippo pathway mutants.

## Discussion

Ds-Fat signaling controls organ shape from *Drosophila* to mammals, but the mechanisms involved have remained poorly understood [17–19]. One of the best studied examples of Ds-Fat control of shape is the *Drosophila* wing, which normally has an elongated shape, but becomes more rounded upon mutation or knockdown of genes involved in Ds-Fat signaling [4–6,8]. Pioneering studies suggested that this was mediated through controlling the orientation of mitotic spindles to orient growth along the proximal-distal axis [40,41]. However, the observation that randomizing spindle orientation did not alter wing shape or growth orientation within the wing disc argued against this [42]. Our observation that stress patterns and myosin distribution are altered in *ds* mutant wing discs, together with observations that direct manipulation of cytoskeletal tension modulates wing shape, suggest that Ds-Ft signaling modulates wing shape primarily through regulating tissue tension during larval growth. Elongation of the wing primordia along the proximal–distal axis, which has been revealed by cellular dynamics to include a combination of oriented cell rearrangement, cell shape changes, and cell divisions, is believed to occur in response to normal stress patterns [43]. The effect of Ds-Fat signaling on spindle orientation could thus be understood as an indirect consequence of the altered stress patterns that occur when genes in this pathway are inactivated.

Our results also emphasize that while the wing undergoes dramatic morphogenesis that reshapes it from imaginal disc to wing during pupal development, and Ds and Fat are expressed and actively influence PCP during pupal development [45–47], their main influence on wing shape occurs prior to this, during the larval growth phase. This was confirmed functionally, by temperature shift experiments establishing that *ds* and *fat* are primarily required during larval development to regulate wing shape. In addition, we observed that their effect on the shape of the wing pouch in the larval imaginal disc prefigures their effect on the shape of the adult wing. That is, despite the dramatic reshaping of wing tissue during metamorphosis, the altered shape of the future wing is evident in the larval disc in the relative increase in length of DV boundary (wing circumference) as compared to the length of AP boundary (proportional to wing length). This observation is consistent with the idea that adult wing shape is preprogrammed in larval wing disc morphology [63]. We note that direct examination of pupal wings revealed that *ds* or *fat* knockdown wings elongate less than control wings during initial flattening and elongation of the wing (6–18 h APF). While this could indicate that Ds and Fat also contribute to wing shape during pupal development, it could alternatively be an indirect consequence of overgrowth of the wing hinge, leading to less effective contraction of the hinge and consequently reduced wing elongation. Overgrowth of the wing hinge is a prominent feature of *ds* and *fat* mutants, and occurs due to Hippo pathway-mediated regulation of Wg [27,34], which is a key driver of wing hinge growth [64].

Ds-Fat signaling modulates distinct, Dachs-dependent, downstream processes to regulate growth, PCP and morphogenesis. We found that Ds-Fat alters the shape of the developing wing pouch from the earliest time that it becomes visibly outlined by Wg expression, at early third instar. This effect of Ds-Fat was shared by inactivation of genes that specifically impair Hippo signaling. However, the initial effect of Hippo pathway genes on wing disc shape was lost during disc growth, and the adult wings that formed from discs where these genes were impaired are not as round as *ds* or *fat* mutant wings. Additionally, we observed that while mutation or knockdown of Hippo pathway genes results in rounder wings, they are not as round as *ds* or *fat* mutant or knockdown wings, even when they result in similar levels of wing overgrowth. Thus, we conclude that effects on Hippo signaling contribute to, but are not sufficient to explain, the effect of Ds-Fat on wing shape. This conclusion is further supported by the observation that wing shape in animals with compromised Hippo signaling in the wing remains sensitive to decreases in cytoskeletal tension generated by *rok* RNAi, whereas *ds* wing shape was not further altered by *rok* RNAi. Ds-Ft signaling also connects to canonical PCP, through physical interaction of Ds and Dachs with the Sple isoform of Pk-Sple [32,33]. However, as mutation or knock down of core PCP components, including *pk*, has only very minor effects on wing shape [65], this cannot account for the effect of Ds-Fat on wing shape. Finally, Ds-Fat have also been proposed to regulate tissue tension through their effects on localization of Dachs [36,41,45], which is a myosin family protein that can bind F-actin [5,66]. The potential ability of Dachs to affect junctional tension has previously been suggested to contribute to effects of Ds-Fat signaling on the shape of the notum during pupal morphogenesis [36] and the orientation of mitotic spindles in wing discs [41], and we suggest that effects of Dachs on tissue tension are likely to contribute to the influence of Ds-Fat on wing shape. In the notum, however, Dachs was inferred to act directly during pupal morphogenesis, in contrast to observations that Ds-Fat makes its key contributions to wing morphogenesis during larval development.

As Dachs localization is polarized in the developing wing imaginal disc [5], the influence of Ds-Fat signaling may in part depend upon this polarization. Junctional tension and myosin localization in much of the wing is normally higher along circumferential junctions (where Dachs accumulates) than along radial junctions [55,56], and we observed that stress anisotropy and myosin polarization in the proximal wing is reduced in the absence of Ds. However, we also see global effects on the overall distribution of tissue tension contributing to shaping the developing wing. This is emphasized first by the broad changes in myosin distribution and tissue tension revealed by laser cutting and imaging and quantitation of myosin levels. The importance of tissue wide effects on tension is also emphasized by the non-autonomous nature of the effects of *dachs* on growth orientation. Thus, while marked clones of cells within *dachs* mutant discs lack normal growth orientation, clones of cells mutant for *dachs* in otherwise wild-type discs exhibit normal growth orientation [5,41]. The observation that wings can be made rounder by broadly decreasing cytoskeletal tension, and more elongated by increasing cytoskeletal tension, also argue for a contribution of global tension patterns to wing shape. Nonetheless, the observation that influence of *ds* on wing shape could only be partially reversed by a broad increase in tension suggests that the normal patterns and polarization of tension within the developing wing disc are also important for control of shape. Finally, we emphasize that the observation that Ds-Fat signaling controls wing morphogenesis through regulation of tissue tension may also apply to other contexts where Ds-Fat signaling controls morphogenesis.

## Materials and methods

### *Drosophila* genetics

All flies were kept on standard cornmeal fly food supplemented with yeast and agar. Stocks were maintained at room temperature. Fly crosses were performed at 25 °C unless otherwise specified. The stocks that were used in this study include: *w*^*1118*^(control), *ds*^*UA071*^ [67], *ds*^*36D*^ [68], *RNAi-ds* (vdrc 36219), *RNAi-ds* (vdrc 4313), GS-ds, *d*^*GC13*^ and *d*^*210*^ [5], *ft*^*G-rv*^ (BDSC#1894), *ft*^*8*^ (BDSC#44257), *RNAi-fat* (vdrc#9396), UAS-fat [62], *ex*^*e1*^ (BDSC#44249), *RNAi-hpo* (BDSC#33614), *wts*^*P2*^ [69], *wts*^*X1*^ [70], *RNAi-wts* (BDSC#41899), UAS- wts [58], Ecad:GFP [36,71], sqh-sqh:GFP [72], *nub-Gal4* [58,73], *hh-Gal4* [44], tub-Gal80^ts^ (BDSC#7017), UAS-dcr2 [74], *RNAi-rok* (vdrc104675), UAS-rok^CA^ (BDSC#6668), Raeppli-CAAX[67E] (BDSC#55083), UAS-InR^act^ (BDSC#8263), *UAS-Zyx:Dachs-V5* [60], UAS-mCD8:RFP (gift of G. Morata, Universidad Autónoma de Madrid, Madrid). *inv-BFP ap-BFP* were created by cloning enhancer sequences driving wing imaginal disc expression of *inv* (R88c04) and *ap* (R42A06) [75] upstream of 2xmTagBFP in place of Gal4 in pBPGUw (addgene 17575) and isolating third chromosome insertions.

### Temperature shift experiments

For temporal knockdown experiments, we used the *nub-GAL4* driver line in combination with *tub-Gal80*^*ts*^ crossed to *RNAi-ft* (vdrc 9396) or *RNAi-ds* (*vdrc 36219*). Crosses and progeny were kept at either 18 °C or 29 °C, following the temperature shift scheme described in Fig 1. Late L3 shifts were done on wandering (wall-crawling) third instar larvae which begins ~12 h before puparium formation, and early pupal shifts were done at 0–8 h APF. For Fat immunostaining larvae were shifted to 18 °C 24 h before puparium formation and stained at 4 h APF.

### Adult wing imaging and analysis

Adult male wings were dissected in isopropanol and mounted in 4:1 Canada Balsam:Methyl Salicylate and imaged using a Zeiss Axioplan2 microscope and a Progress camera. The major and minor axes, as well as the area of adult wings, were measured by manually tracing digital wing images by using ‘Free hand selection tool’ in Fiji [76] with ‘Fit Ellipse’ ‘Area’ and ‘Shape Descriptors’ options selected in ‘Set measurement tool’ selections.

### Pupal wing imaging

For pupal wing imaging, larvae of the desired genotype were scored at the late third instar stage and kept at 25 °C, scored as 0 h After puparium formation (APF) when they formed white prepupae and imaged at 6, 12 and 18 h APF. Prior to imaging at each stage, pupae were gently removed from vials using a moistened brush (Princeton Velvetouch Series 3950 Synthetic Round Brush, Size 1) and aligned on a glass slide with one pupal wing oriented toward the objective. Pupae were aligned using a stereo microscope (Zeiss Stemi SV11 Apo) equipped with fluorescence illumination (Excelitas X-Cite Series 120Q). Once aligned, the pupae adhered to the slide within a few seconds and were ready for imaging. Pupal wings were imaged through the pupal case using a Leica TCS SP8 Confocal Microscope.

### Immunostaining and microscopy

To obtain wing discs at different stages, the flies were transferred to new vials for 4–6 h, and larvae were dissected at 72, 84, 96, 108 and 120 h After Egg Laying (AEL). For most experiments, wing discs were fixed for 15 min in 4% paraformaldehyde at room temperature (RT), whereas Sqh:GFP discs were fixed for 12 min in 4% paraformaldehyde at RT. Fixed larval tissues were rinsed twice with PBT [1×PBS with 0.1% (v/v) Triton-x-100 + 1% (w/v) BSA + 0.01% (v/v) Na-azide], then washed 3 times 15 min each in PBT at RT, incubated for 30 min in blocking solution [PBT + 5% (v/v) Donkey serum], and incubated with primary antibodies overnight at 4 °C with gentle mixing. Primary antibodies used were rat anti-E-cadherin (Developmental Studies Hybridoma Bank, DCAD2-c; 1:200), mouse anti-Wg (Developmental Studies Hybridoma Bank, 9A4-c; 1:300), rabbit anti-Dcr2 (Abcam, ab4732; 1:1,000), rat anti-Fat ([77]; 1:1,000). Sqh localization was monitored using sqh:GFP [72]. Discs were then rinsed twice with PBT, then washed 4 times 15 min each in PBT, incubated for 30 min in blocking solution, and incubated with secondary antibodies for 2 hours at RT with gentle mixing, with tubes wrapped in aluminum foil. Secondary antibodies were from Jackson ImmunoResearch Laboratories, Invitrogen and Biotium (20137). Discs were then rinsed twice with PBT, washed 2 times 15 min in PBT, incubated 15 min with Hoechst 33342 (Invitrogen, H3570) to label DNA, rinsed twice with PBT, then washed 2 times 15 min each in PBT. Wing imaginal disc were dissected using fine forceps, and mounted onto a microscope slide in Vectashield (Vector Laboratories, H-1000). Images were collected on the Leica SP8 confocal microscope. Original image files are available at the Bioimage archive: https://doi.org/10.6019/S-BIAD3480.

### Wing pouch shape analysis

To calculate the dorsal–ventral axis (D–V)- anterior–posterior axis (A–P) ratio for wing pouch shape analysis, the wing discs were stained with mouse anti-Wg, and the wing pouch was defined by the inner ring of Wg expression. The D–V boundary and an approximate A–P axis were manually traced inside the inner ring of Wg expression using the ‘Freehand Line’ tool in Fiji in 2D projections of confocal image stacks through wing discs that were slightly flattened by a coverslip. The aspect ratio of the wing pouch was calculated by dividing the length of the D–V axis by the length of the A–P axis. For 3D measurements of wing pouch shape, wing discs were mounted with glass beads as spacers to preserve normal disc curvature, with 100 µm glass beads (Duke Scientific Corporation, #9100) used for 120 h wing discs and 50 µm glass beads (Electron Microscopy Sciences, #02726AB) used for 72 h wing discs. For 3D measurement along the A–P boundary, the Freehand Line tool in Fiji was used to trace the surface of the disc from the dorsal to the ventral side of the Wg inner ring in a Z slice along the center of the wing pouch. For 3D measurement of the D–V length, the filament creation tool in IMARIS 11 was used to define and measure the length of Wg expression along the D–V boundary between the inner ring of Wg hinge expression. We note that as the D–V length corresponds approximately to the wing circumference and the A–P length corresponds to twice the wing length (due to folding of the wing along the D–V boundary), geometrically the measurement we report corresponds roughly to *C*/2*d*, where *C* = circumference and *d* = diameter. For a perfect circle, this ratio would = *π*/2 (i.e., ~1.57). For an elongated elliptical shape, where *d* is the major axis, the ratio gets smaller as it gets more elliptical (for an infinitely long and narrow ellipse *C* would = 2*d*, so the *C*/2*d* ratio would = 1). Conversely, for an ellipse where *d* is the minor axis, the ratio would be greater than *π*/2. The measured numbers are skewed by the fact that we are not actually measuring these perfect geometric shapes, and the wing elongates during pupal development, but the mathematical principle that the ratio gets smaller as the wing gets more elongated and larger as it gets wider remains valid.

### Raeppli clones

To generate Raeppli clones, *nub-Gal4 UAS-dcr2 > Raeppli-CAAX-67E* flies were crossed with either *w*^*1118*^ (control), *RNAi-rok*, or *RNAi-fat* and then maintained at 25 °C. Larvae were heat-shocked in a water bath at 36 °C for 8 min between 60 and 72 hours AEL, then transferred to 29 °C. Larvae were dissected 36 hours after heat shock to analyze Raeppli clones. To quantify Raeppli clone shape, clone boundaries were manually traced and analyzed using the Shape Descriptors function in Fiji/ImageJ.

### Statistical analysis

Statistical significance was examined using GraphPad Prism software by performing Student *t* test for comparisons between two groups or one-way ANOVA for multiple groups, with *p* < 0.05 considered statistically significant. A Kolmogorov-Smirnov test was used for comparing myosin polarity distributions. All quantifications are presented as the mean ± SD. For all statistical tests, ns indicates *P* > 0.05, * indicates *P* ≤ 0.05, ** indicates *P* ≤ 0.01, *** indicates *P* ≤ 0.001, **** indicates *P* ≤ 0.0001.

### Image analysis

For the Sqh:GFP fluorescence intensity heat map, confocal image stacks were processed using a custom MATLAB script [78,79] to project the apical surface onto a 2D plane, based on the maximal brightness of E-Cadherin. The intensity of the channel of interest (Sqh:GFP) is normalized over the reference channel (E-cad) intensity and represented by the heat map. To quantify cell shapes, confocal image stacks were processed using a custom MATLAB script [78,79] to generate flattened projections. The Tissue Analyzer plugin in Fiji was then used to segment individual cells based on the E-cad signal. Cell eccentricity and polarity of myosin were calculated using Quantify Polarity software [80]. Polarity angles were graphed in a Rose plot using MATLAB.

### Live imaging and laser ablation

For circular ablation experiments, wing discs at 108 h were dissected and cultured in live imaging media by following the protocol established by Dye and colleagues [43]. This uses Grace′s Insect Medium (With L-glutamine, without sodium bicarbonate) (Sigma-Aldrich, G9771) dissolved in water with pH adjusted to 6.6–6.7 and filter sterilized (Thermo Scientific # 450-0020). Prior to each experiment, the Grace′s Insect Medium stock solution was supplemented with 5% fetal bovine serum (Thermo Fisher, 10082147), 100× penicillin-streptomycin (Thermo Fisher, #15070063), and 10 nM 20-hydroxy-ecdysone (Sigma, H5142). Wing disc dissection and mounting was done as described previously [42,81]. Circular ablation was performed using an Andor Dragonfly500 Spinning Disk Confocal with a MicroPoint ablation system. Circles of 10 μm radius were cut using Micropoint settings of 60% Laser power, Repetition rate 16 Hz, and 2 Repeats. Roundness and maximum expansion area were measured 1 min after ablation using Fiji/ImageJ. Each ablation was performed on a separate wing disc. Graphs were made using GraphPad Prism, and images were arranged in Adobe Illustrator. To measure recoil velocity, cell junctions in proximal (tangential junctions) and D–V boundary (parallel to the D–V boundary) regions in wing imaginal discs from control and *ds* mutants were ablated using a Micropoint pulsed laser system tuned to 365 nm with 60% laser power, repetition rate 16 Hz and 2 repeats, on an Andor Dragonfly500 Spinning Disk Confocal. Discs were imaged every second and based on velocity graphs the displacement of vertices over the first two second after ablation was used to calculate the recoil velocities.

## Supporting information

S1 Data Individual numerical values for data presented in figures. This file includes the individual numerical values obtained and graphed in Figs 1D, 1S, 2E, 2O, 3D, 4F–4I, 5F, 5I′, 5J’, 6D, 6H, 6L, 7G, 7L, 7M, 8G, 8K–8N, S2E, S3I–S3L, S4D, S4E, S4G, S5E, S5H, S5I, S6A–S6D, S6J, S7F, S7G, S7J, and S7K. Descriptions of how these data are plotted are contained within the legends of each of these figures.
(XLSX)

S1 Fig Examination of Fat recovery when Fat knockdown is suppressed by shifting animals to 18 °C. 4hAPF pupal wings from *nub-Gal4 UAS-RNAi-fat* at 18 °C (control, **A**), 29 °C until 6 h before puparium formation, then shifted to 18 °C **(B)**, 29 °C until 24 h before puparium formation, then shifted to 18 °C **(C)**, immunostained with rat anti-Fat antibody. Scale bar: 50µm.
(PDF)

S2 Fig Examination of larvel wing pouch shape in 3D. **(A–D)** Confocal micrographs of wing disc stained for Wg from **(A, C)** *control* at 72 h AEL (A, *n* = 7), 120 h AEL (C, *n* = 11), *ds*^*UA071*^*/ds*^*36D*^ at 72 h AEL (B, *n* = 10), 120 h AEL (D, *n* = 11). Panels at right and bottom YZ and XZ show slices across the length and width of the disc, and include staining for DNA, Wg and E-cad to show the folds at 120 h. Red arrows point to the inner Wg ring at 120 h in YZ slices along A–P boundary. **(E)** Histogram quantifying shape in 3D shape. Error bar indicates mean ± s.d., the significance of differences relative to *control* at same developmental stage, calculated by *t* test, is indicated by black asterisks. An example of the A–P length measure including folds at 120 h is indicated by white line at right in panel C. The data underlying the results presented in E is available in S1 Data.
(PDF)

S3 Fig Influence of Hippo signaling on shape of larval wing pouch and adult wing. **(A–D)** Male wings from *w*^*1118*^ (A; *n* = 35), *nub-GAL4:UAS-Dcr2/ UAS-RNAi-wts* (B; *n* = 22), *ex*^*e1*^*/ ex*^*e1*^ (C; *n* = 11) and *nub-GAL4:UAS-Dcr2/ UAS-RNAi-hpo* (D; *n* = 13). Scale bar = 500 µm. **(E–H)** Wing discs stained for Wg from **(E)** *wts*^*P2*^/*wts*^*X1*^at 72 h AEL (*n* = 7), 84 h AEL (*n* = 5), 96 h AEL (*n* = 8), 108 h AEL (*n* = 5), and 120 h AEL (*n* = 5), **(F)** *ex*^*e1*^*/ ex*^*e1*^ at 72 h AEL (*n* = 11), 84 h AEL (*n* = 9), 96 h AEL (*n* = 11), 108 h AEL (*n* = 9) and 120 h AEL (*n* = 11), **(G)** *nub-GAL4:UAS-Dcr2/ UAS-RNAi-wts* at 72 h AEL (*n* = 9), 84 h AEL (*n* = 6), 96 h AEL (*n* = 5), 108 h AEL (*n* = 12) and 120 h AEL (*n* = 13), **(H)** *nub-GAL4:UAS-Dcr2/ UAS-RNAi-hpo* at 72 h AEL (*n* = 12), 84 h AEL (*n* = 16), 96 h AEL (*n* = 12), 108 h AEL (*n* = 15) and 120 h AEL (*n* = 12). Scale bar = 100 µm. **(I)** Histogram quantifying shape for adult wings described in A–D. Error bar indicates mean ± s.d., the significance of differences relative to *w*^*1118*^, calculated by one-way ANOVA, is indicated by asterisks. **(J, K)** Histograms quantifying wing pouch shape for discs described in E-H. Error bar indicates mean ± s.d., the significance of differences relative to *w*^*1118*^ at same developmental stage (J), and relative to *nub-GAL4:UAS-Dcr2* control at same developmental stage (K) calculated using one-way ANOVA, is indicated by asterisks or ns. Control data used here for comparison is as of shown in Fig 3 for J and in Fig 5 for K. **(L)** Histogram showing relative wing area for adult male wings of the indicated genotypes, normalized to the mean control value. Error bar indicates mean ± s.d., the significance of differences relative to control, calculated using one-way ANOVA, is indicated by asterisks. The data underlying the results presented in I, J, K, and L are available in S1 Data.
(PDF)

S4 Fig Influence of InR activation on wing shape. **(A–C)** Male wings from *nub-GAL4:UAS-Dcr2/+* as control (A; *n* = 28), *nub-GAL4:UAS-Dcr2/UAS-InR*^*act*^ (B; *n* = 10), *nub-GAL4:UAS-Dcr2/ UAS-RNAi-fat* (C; *n* = 12). Scale bar = 500 µm. **(D, E)** Histograms quantifying (D) wing area (E) wing shape, compared to *nub-GAL4:UAS-Dcr2/+*. Error bars indicate mean ± s.d., the significance of differences relative to *nub-GAL4:UAS-Dcr2/+* is indicated by black asterisks and was calculated using one-way ANOVA on measurements from the number of adult wings indicated above and the significance of difference between *UAS-InR*^*act*^ and *UAS-RNAi-fat* is indicated by green symbols. **(F)** Wing discs stained for Wg from *nub-GAL4:UAS-Dcr2/UAS-InR*^*act*^ at 72 h AEL (*n* = 7), 84 h AEL (*n* = 8), 96 h AEL (*n* = 8), 108 h AEL (*n* = 16), and 120 h AEL (*n* = 9). Scale bar = 100 µm. **(G)** Histogram quantifying wing pouch shape. Error bar indicates mean ± s.d., the significance of differences relative to *nub-GAL4:UAS-Dcr2/+* from the same developmental stage is indicated by ns/black asterisks and was calculated using t-tests on measurements from the number of samples indicated above. Control data used here for comparison is the same as shown in Fig 6. The data underlying the results presented in D, E and G are available in S1 Data.
(PDF)

S5 Fig Influence of Fat overexpression or *dachs* mutant on wing shape. **(A–D)** Male wings from *nub-GAL4:UAS-Dcr2/+* (A; *n* = 28), *nub-GAL4:UAS-Dcr2/UAS-fat* (B; *n* = 19), *nub-GAL4:UAS-Dcr2/GS-ds* (C; *n* = 8), and *nub-GAL4:UAS-Dcr2/UAS-wts* (D; *n* = 9). Scale bar = 500 µm. **(E)** Histogram quantifying shape for wings described in A-D. Error bars indicate mean ± s.d., the significance of differences relative to *nub-GAL4:UAS-Dcr2/+*, calculated by one-way ANOVA, is indicated by asterisks. **(F, G)** Wing discs stained for Wg **(F)** from *nub-GAL4:UAS-Dcr2/UAS-ft* at 72 h AEL (*n* = 10), 84 h AEL (*n* = 8), 96 h AEL (*n* = 11), 108 h AEL (*n* = 11), and 120 h AEL (*n* = 11) **(G)** *d*^*210*^*/ d*^*GC13*^ at 72 h AEL (*n* = 10), 84 h AEL (*n* = 10), 96 h AEL (*n* = 10), 108 h AEL (*n* = 11), and 120 h AEL (*n* = 11). Scale bar = 100 µm. **(H, I)** Histograms quantifying wing pouch shape for F and G, respectively. Error bars indicate mean ± s.d., the significance of differences relative to *nub-GAL4:UAS-Dcr2/+*. Control data used here for comparison is the same as shown in Fig 6 for H and Fig 3 for I. The data underlying the results presented in E, H and I are available in S1 Data.
(PDF)

S6 Fig Additional analysis of stress patterns and tension in *ds* mutant wing discs. **(A–D)** Histograms, based on data described in Fig 4, comparing the shape (A, B) and size (C, D) of cut regions at their maximal expansion between D–V boundary and proximal regions. Error bar indicates mean ± s.d., the significance of differences is indicated by black asterisks and were calculated using *t*-tests. **(E–H)** High magnification views of the wing imaginal discs, 1 s before and 2 s after laser cutting of cell junctions between the red arrows. **(I)** Wing disc schematic showing where junctions were cut, in the D–V boundary region junctions oriented parallel to the D–V boundary were measured; in proximal regions tangential junctions were measured. **(J)** Quantitation of initial recoil velocities of vertices adjacent to cut junctions, *N* = 20 proximal junctions and 12 junctions at D–V boundary from 14 wing discs for control; *N* = 14 proximal junctions and 19 junctions at D–V boundary from 11 wing discs for *ds*. The significance of differences in recoil velocity between control and ds mutant discs is indicated by asterisks. The data underlying the results presented in A, B, C, D and J are available in S1 Data.
(PDF)

S7 Fig Influence and interactions between cytoskeletal tension and Hippo pathway knockdown. **(A–D)** Male wings from *ex*^*e1*^ (A; *n* = 11), *nub-Gal4 UAS-RNAi-rok: ex*^*e1*^ (B; *n* = 11), *nub-Gal4 UAS-RNAi-hpo* (C; *n* = 13), and *nub-Gal4 UAS-RNAi-rok*, *UAS-RNAi-hpo* (D; *n* = 7). Scale bar = 500 µm. **(E)** Wing discs stained for Wg from *nub-Gal4 UAS-RNAi-rok: ex*^*e1*^ at 72 h AEL (*n* = 5), 84 h AEL (*n* = 6), 96 h AEL (*n* = 8), 108 h AEL (*n* = 10), and 120 h AEL (*n* = 8). Scale bar = 100 µm. **(F)** Histogram quantifying shape for wings described in A-D. Error bar indicates mean ± s.d., the significance of differences relative to control, calculated by one-way ANOVA, is indicated by black asterisks, and differences between presence or absence of *UAS-RNAi-rok* is indicated by green asterisks. **(G)** Histogram quantifying wing pouch shape for (E). Error bar indicates mean ± s.d., the significance of differences relative to control (control data from Fig 3, *ex*^*e1*^ data from S1 Fig) is indicated by black symbols and was calculated using t-tests on measurements from the number of samples indicated above. Comparison between the with and without *UAS-*RNAi-rok sets was done by *t* test, and the significance is color-coded by time point. **(H)** Wing disc expressing Sqh:GFP at 108 h from *Sqh:GFP/+; ex*^*e1*^. **(I)** Wing disc expressing Sqh:GFP at 108 h and *UAS-RNAi-hpo* under hh-Gal4 control. Panels marked prime shoe heat maps of Sqh:GFP intensity normalized to E-cad; Scale for the heat map is at top right. **(J, K**) Graphs of relative Sqh intensity in proximal or D–V boundary regions for control and RNAi *hpo* discs, from locations indicated in Fig 5. Error bar indicates mean ± s.d., and significance of differences is indicated by asterisks. The data underlying the results presented in F, G, J, and K are available in S1 Data.
(PDF)

## Abbreviations

AEL: After Egg Laying
Ds: Dachsous
Fj: four-jointed
PCP: planar cell polarity
Rok: Rho-associated kinase
RT: room temperature
