# Supplementary figures and images for: Dachsous-Fat signaling shapes the Drosophila wing through mechanical forces
Source: PLoS Biol. 2026 Jul 14;24(7):e3003883. doi: 10.1371/journal.pbio.3003883 (PMC13375139; doi:10.1371/journal.pbio.3003883)

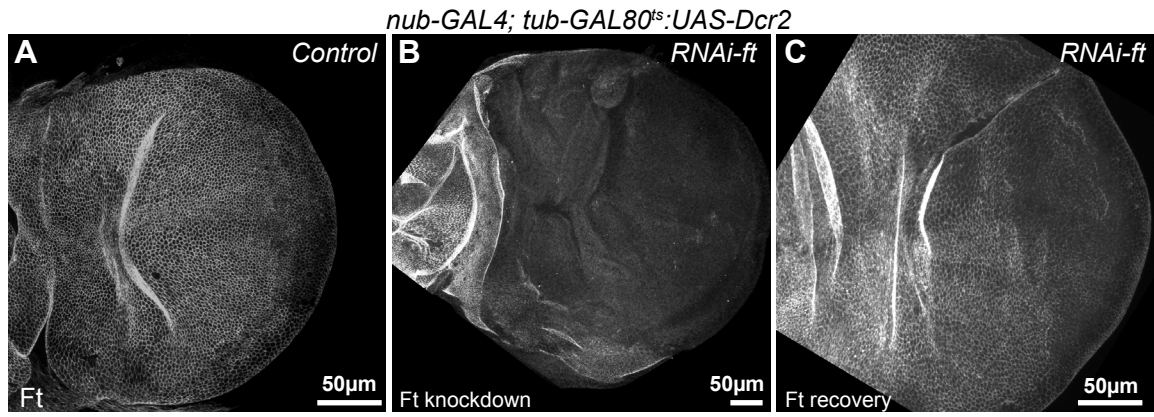

Supplement: S1 Fig — 4hAPF pupal wings from nub-Gal4 UAS-RNAi-fat at 18 °C (control, A), 29 °C until 6 h before puparium formation, then shifted to 18 °C (B), 29 °C until 24 h before puparium formation, then shifted to 18 °C (C), immunostained with rat anti-Fat antibody. Scale bar: 50µm. (PDF) [file pbio.3003883.s002.pdf]

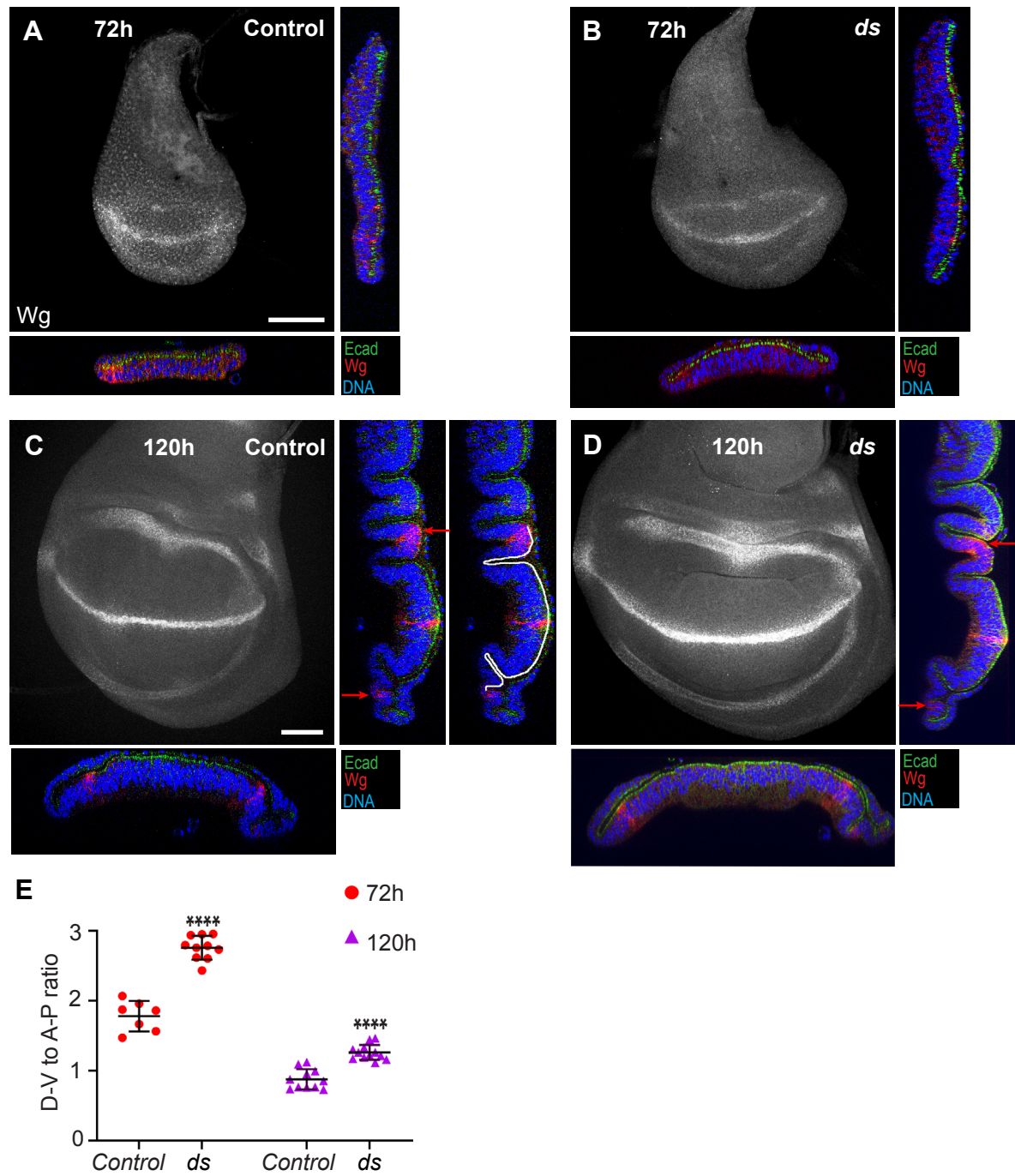

Supplement: S2 Fig — (A–D) Confocal micrographs of wing disc stained for Wg from (A, C) control at 72 h AEL (A, n = 7), 120 h AEL (C, n = 11), dsUA071/ds36D at 72 h AEL (B, n = 10), 120 h AEL (D, n = 11). Panels at right and bottom YZ and XZ show slices across the length and width of the disc, and include staining for DNA, Wg and E-cad to show the folds at 120 h. Red arrows point to the inner Wg ring at 120 h in YZ slices along A–P boundary. (E) Histogram quantifying shape in 3D shape. Error bar indicates mean ± s.d., the significance of differences relative to control at same developmental stage, calculated by t test, is indicated by black asterisks. An example of the A–P length measure including folds at 120 h is indicated by white line at right in panel C. The data underlying the results presented in E is available in S1 Data. (PDF) [file pbio.3003883.s003.pdf]

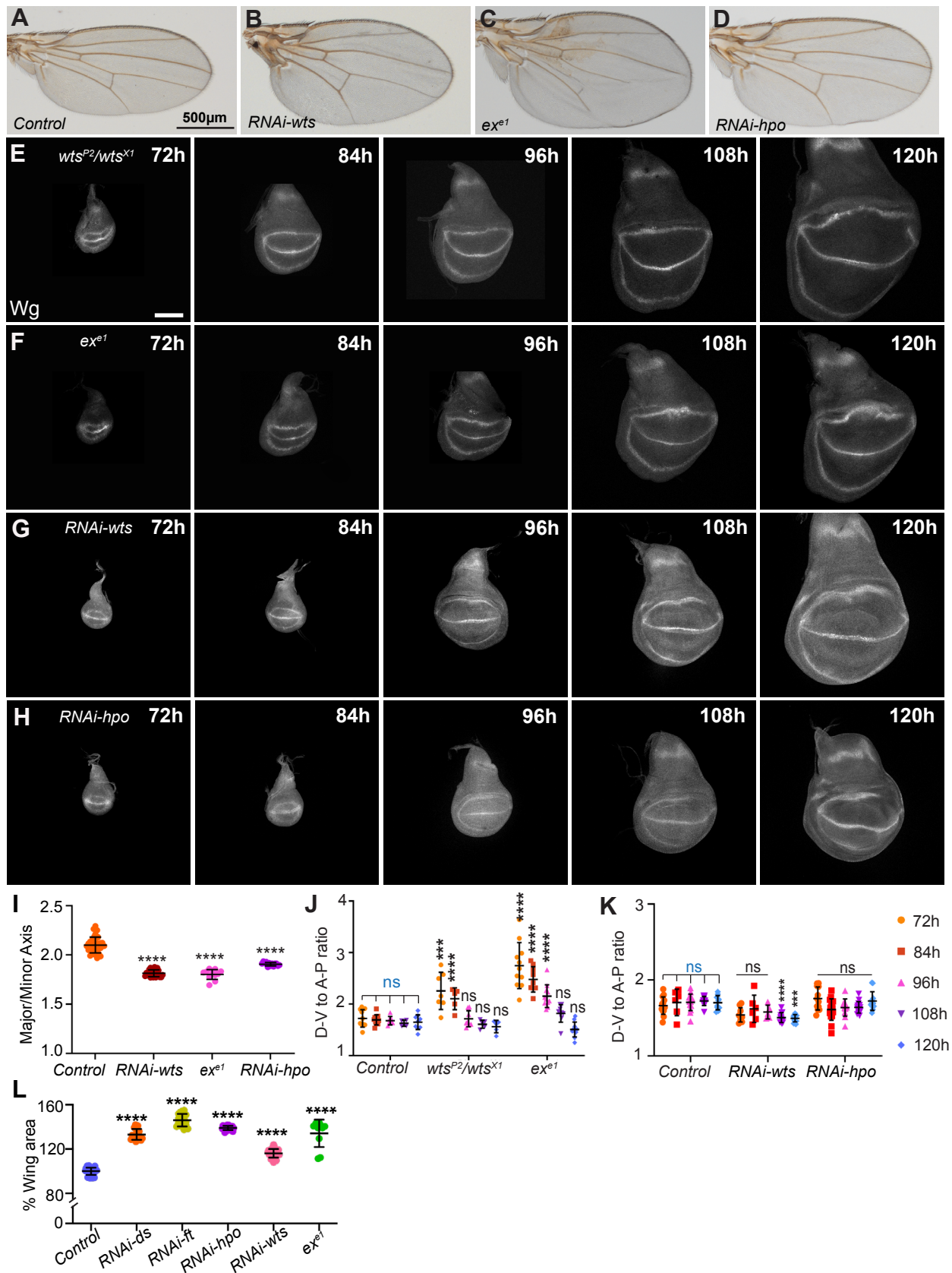

Supplement: S3 Fig — (A–D) Male wings from w1118 (A; n = 35), nub-GAL4:UAS-Dcr2/ UAS-RNAi-wts (B; n = 22), exe1/ exe1 (C; n = 11) and nub-GAL4:UAS-Dcr2/ UAS-RNAi-hpo (D; n = 13). Scale bar = 500 µm. (E–H) Wing discs stained for Wg from (E) wtsP2/wtsX1at 72 h AEL (n = 7), 84 h AEL (n = 5), 96 h AEL (n = 8), 108 h AEL (n = 5), and 120 h AEL (n = 5), (F) exe1/ exe1 at 72 h AEL (n = 11), 84 h AEL (n = 9), 96 h AEL (n = 11), 108 h AEL (n = 9) and 120 h AEL (n = 11), (G) nub-GAL4:UAS-Dcr2/ UAS-RNAi-wts at 72 h AEL (n = 9), 84 h AEL (n = 6), 96 h AEL (n = 5), 108 h AEL (n = 12) and 120 h AEL (n = 13), (H) nub-GAL4:UAS-Dcr2/ UAS-RNAi-hpo at 72 h AEL (n = 12), 84 h AEL (n = 16), 96 h AEL (n = 12), 108 h AEL (n = 15) and 120 h AEL (n = 12). Scale bar = 100 µm. (I) Histogram quantifying shape for adult wings described in A–D. Error bar indicates mean ± s.d., the significance of differences relative to w1118, calculated by one-way ANOVA, is indicated by asterisks. (J, K) Histograms quantifying wing pouch shape for discs described in E-H. Error bar indicates mean ± s.d., the significance of differences relative to w1118 at same developmental stage (J), and relative to nub-GAL4:UAS-Dcr2 control at same developmental stage (K) calculated using one-way ANOVA, is indicated by asterisks or ns. Control data used here for comparison is as of shown in Fig 3 for J and in Fig 5 for K. (L) Histogram showing relative wing area for adult male wings of the indicated genotypes, normalized to the mean control value. Error bar indicates mean ± s.d., the significance of differences relative to control, calculated using one-way ANOVA, is indicated by asterisks. The data underlying the results presented in I, J, K, and L are available in S1 Data. (PDF) [file pbio.3003883.s004.pdf]

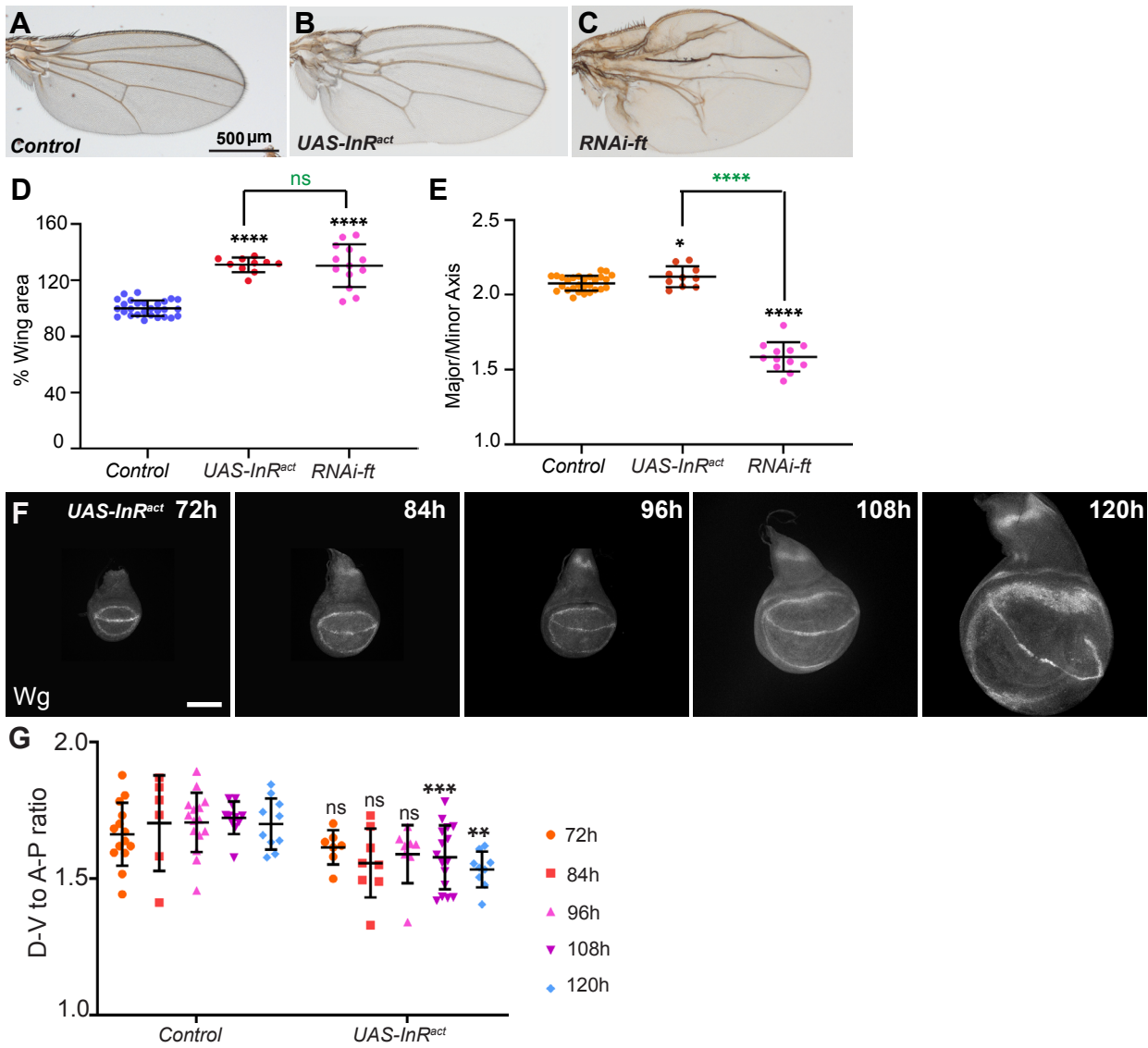

Supplement: S4 Fig — (A–C) Male wings from nub-GAL4:UAS-Dcr2/+ as control (A; n = 28), nub-GAL4:UAS-Dcr2/UAS-InRact (B; n = 10), nub-GAL4:UAS-Dcr2/ UAS-RNAi-fat (C; n = 12). Scale bar = 500 µm. (D, E) Histograms quantifying (D) wing area (E) wing shape, compared to nub-GAL4:UAS-Dcr2/+. Error bars indicate mean ± s.d., the significance of differences relative to nub-GAL4:UAS-Dcr2/+ is indicated by black asterisks and was calculated using one-way ANOVA on measurements from the number of adult wings indicated above and the significance of difference between UAS-InRact and UAS-RNAi-fat is indicated by green symbols. (F) Wing discs stained for Wg from nub-GAL4:UAS-Dcr2/UAS-InRact at 72 h AEL (n = 7), 84 h AEL (n = 8), 96 h AEL (n = 8), 108 h AEL (n = 16), and 120 h AEL (n = 9). Scale bar = 100 µm. (G) Histogram quantifying wing pouch shape. Error bar indicates mean ± s.d., the significance of differences relative to nub-GAL4:UAS-Dcr2/+ from the same developmental stage is indicated by ns/black asterisks and was calculated using t-tests on measurements from the number of samples indicated above. Control data used here for comparison is the same as shown in Fig 6. The data underlying the results presented in D, E and G are available in S1 Data. (PDF) [file pbio.3003883.s005.pdf]

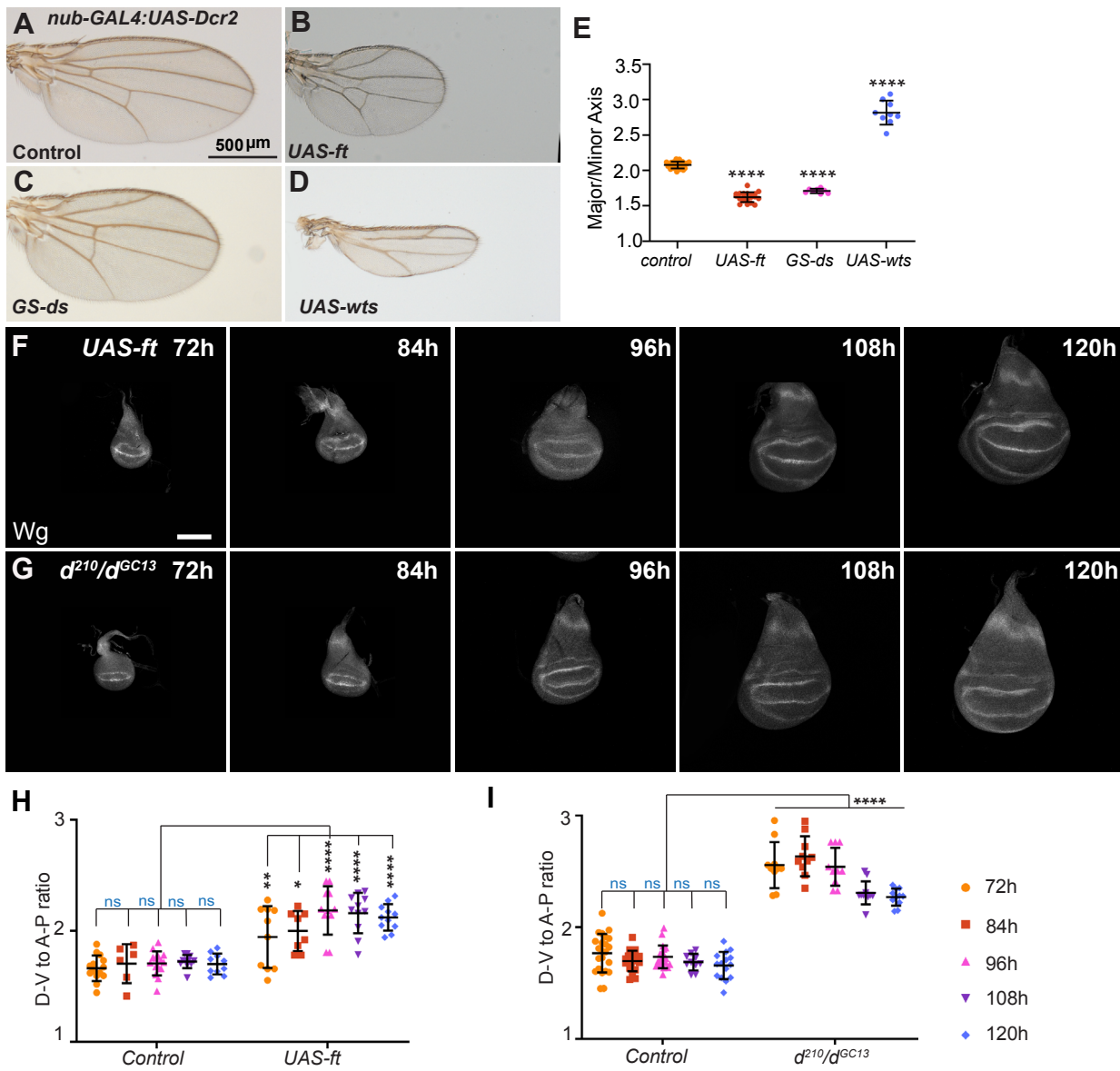

Supplement: S5 Fig — (A–D) Male wings from nub-GAL4:UAS-Dcr2/+ (A; n = 28), nub-GAL4:UAS-Dcr2/UAS-fat (B; n = 19), nub-GAL4:UAS-Dcr2/GS-ds (C; n = 8), and nub-GAL4:UAS-Dcr2/UAS-wts (D; n = 9). Scale bar = 500 µm. (E) Histogram quantifying shape for wings described in A-D. Error bars indicate mean ± s.d., the significance of differences relative to nub-GAL4:UAS-Dcr2/+, calculated by one-way ANOVA, is indicated by asterisks. (F, G) Wing discs stained for Wg (F) from nub-GAL4:UAS-Dcr2/UAS-ft at 72 h AEL (n = 10), 84 h AEL (n = 8), 96 h AEL (n = 11), 108 h AEL (n = 11), and 120 h AEL (n = 11) (G) d210/ dGC13 at 72 h AEL (n = 10), 84 h AEL (n = 10), 96 h AEL (n = 10), 108 h AEL (n = 11), and 120 h AEL (n = 11). Scale bar = 100 µm. (H, I) Histograms quantifying wing pouch shape for F and G, respectively. Error bars indicate mean ± s.d., the significance of differences relative to nub-GAL4:UAS-Dcr2/+. Control data used here for comparison is the same as shown in Fig 6 for H and Fig 3 for I. The data underlying the results presented in E, H and I are available in S1 Data. (PDF) [file pbio.3003883.s006.pdf]

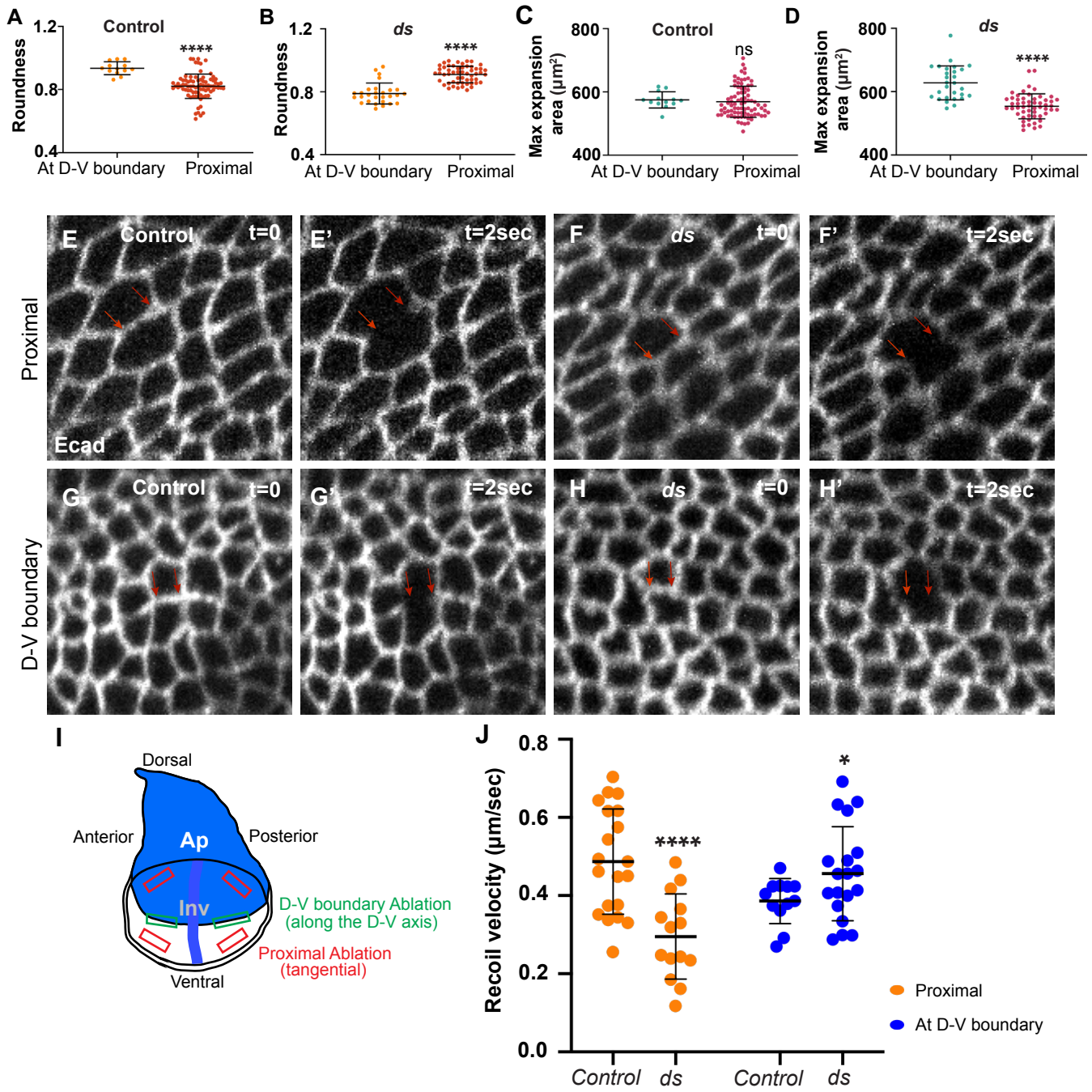

Supplement: S6 Fig — (A–D) Histograms, based on data described in Fig 4, comparing the shape (A, B) and size (C, D) of cut regions at their maximal expansion between D–V boundary and proximal regions. Error bar indicates mean ± s.d., the significance of differences is indicated by black asterisks and were calculated using t-tests. (E–H) High magnification views of the wing imaginal discs, 1 s before and 2 s after laser cutting of cell junctions between the red arrows. (I) Wing disc schematic showing where junctions were cut, in the D–V boundary region junctions oriented parallel to the D–V boundary were measured; in proximal regions tangential junctions were measured. (J) Quantitation of initial recoil velocities of vertices adjacent to cut junctions, N = 20 proximal junctions and 12 junctions at D–V boundary from 14 wing discs for control; N = 14 proximal junctions and 19 junctions at D–V boundary from 11 wing discs for ds. The significance of differences in recoil velocity between control and ds mutant discs is indicated by asterisks. The data underlying the results presented in A, B, C, D and J are available in S1 Data. (PDF) [file pbio.3003883.s007.pdf]

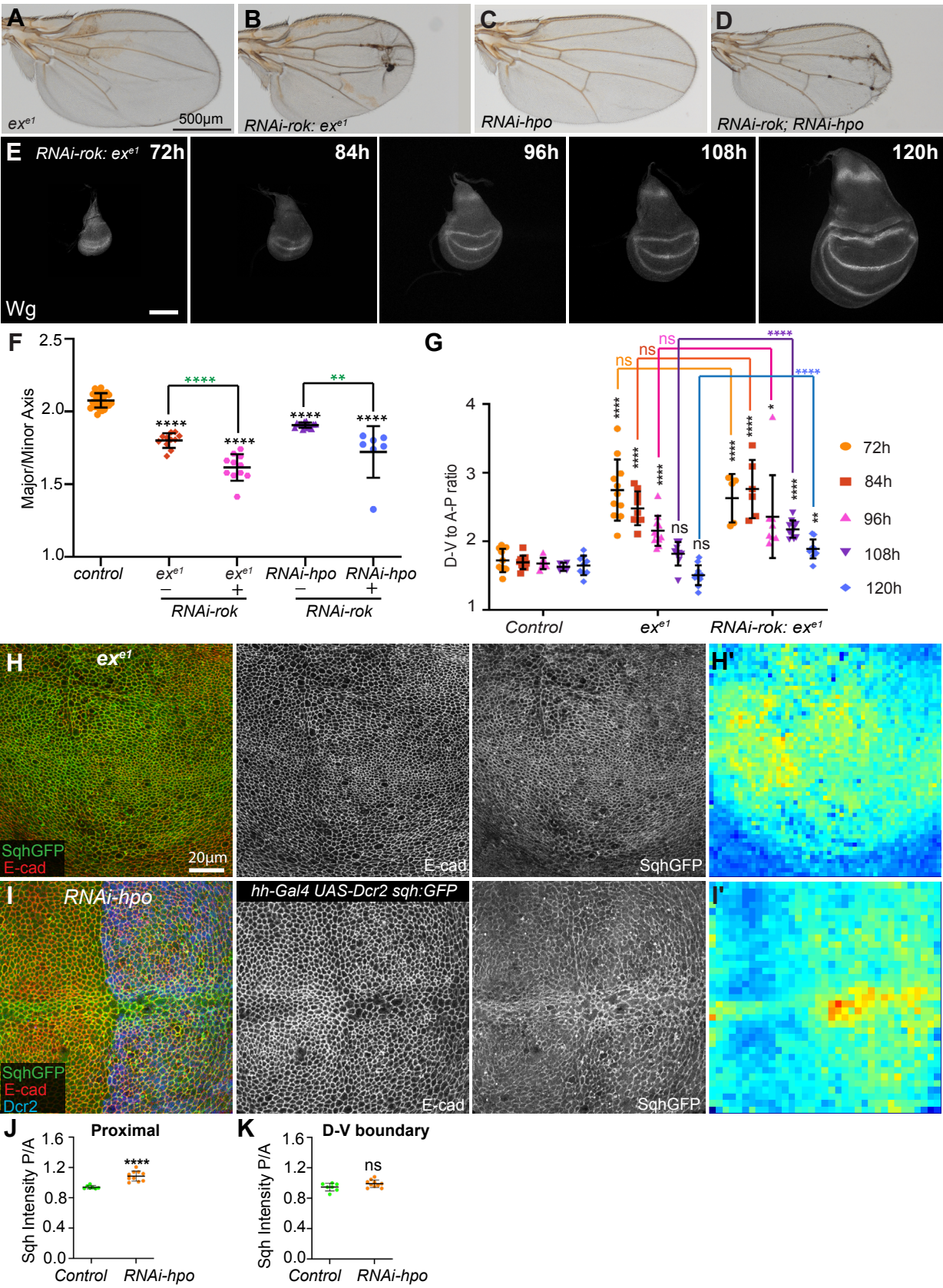

Supplement: S7 Fig — (A–D) Male wings from exe1 (A; n = 11), nub-Gal4 UAS-RNAi-rok: exe1 (B; n = 11), nub-Gal4 UAS-RNAi-hpo (C; n = 13), and nub-Gal4 UAS-RNAi-rok, UAS-RNAi-hpo (D; n = 7). Scale bar = 500 µm. (E) Wing discs stained for Wg from nub-Gal4 UAS-RNAi-rok: exe1 at 72 h AEL (n = 5), 84 h AEL (n = 6), 96 h AEL (n = 8), 108 h AEL (n = 10), and 120 h AEL (n = 8). Scale bar = 100 µm. (F) Histogram quantifying shape for wings described in A-D. Error bar indicates mean ± s.d., the significance of differences relative to control, calculated by one-way ANOVA, is indicated by black asterisks, and differences between presence or absence of UAS-RNAi-rok is indicated by green asterisks. (G) Histogram quantifying wing pouch shape for (E). Error bar indicates mean ± s.d., the significance of differences relative to control (control data from Fig 3, exe1 data from S1 Fig) is indicated by black symbols and was calculated using t-tests on measurements from the number of samples indicated above. Comparison between the with and without UAS-RNAi-rok sets was done by t test, and the significance is color-coded by time point. (H) Wing disc expressing Sqh:GFP at 108 h from Sqh:GFP/+; exe1. (I) Wing disc expressing Sqh:GFP at 108 h and UAS-RNAi-hpo under hh-Gal4 control. Panels marked prime shoe heat maps of Sqh:GFP intensity normalized to E-cad; Scale for the heat map is at top right. (J, K) Graphs of relative Sqh intensity in proximal or D–V boundary regions for control and RNAi hpo discs, from locations indicated in Fig 5. Error bar indicates mean ± s.d., and significance of differences is indicated by asterisks. The data underlying the results presented in F, G, J, and K are available in S1 Data. (PDF) [file pbio.3003883.s008.pdf]
