# Supplementary material for: Dachsous-Fat signaling shapes the Drosophila wing through mechanical forces
Source: PLoS Biol. 2026 Jul 14;24(7):e3003883. doi: 10.1371/journal.pbio.3003883 (PMC13375139; doi:10.1371/journal.pbio.3003883)
Supplement: Attachment — Submitted filename: Revised Response to Reviews_v1.docx [file pbio.3003883.s012.docx]

**Response to Reviewer’s Comments**

We thank all the reviewers and editors for their evaluation and additional comments. The only comment requiring a response is comment 2 of Reviewer 1.

Reviewers' comments
Rev. 1:
Overall, the revision is responsive to my prior comments. The most critical methodological concern is convincingly resolved by the addition of recoil velocity data that corroborates the circular ablation results. The myosin polarity analysis and the expanded Dachs experiments are genuine advances. 

1. I stated the timing of temperature shifts was vague and a late L3 shift-up was missing.
The authors added precise timing definitions and the requested late L3 shift-up experiment. Figure 1R adds the missing shift-up.

2. I argued the shape metric was topologically mislabeled and should be inverted.
The manuscript now clearly states in the Figure 2 legend and main text that the DV length corresponds to the wing circumference and the AP length is proportional to twice the wing length. This is a defensible clarification. However, the authors declined to invert the ratio, which will leave some readers confused since a larger DV/AP ratio in their system corresponds to a rounder wing, the opposite of intuition. Either invert the ratio as I had previously requested or add text explicitly flagging this counter-intuitive metric.

**Response**

We're not sure where the reviewer's intuition comes from. As we explain and the reviewer notes, "the DV length corresponds to the wing circumference and the AP length is proportional to twice the wing length." Thus, geometrically, the measurement we report corresponds to C/2d, where C= circumference and d = diameter. For a perfect circle, this ratio would = π/2 (ie ~ 1.57). For an elliptical shape, where d is the major axis, the ratio is going to get smaller as it gets more elliptical (for an infinitely long and narrow ellipse C would = 2d, so the C/2d ratio would =1). The measured numbers are skewed by the fact that we are not actually measuring these perfect geometric shapes, and the wing elongates during pupal development, but the mathematical intuition nonetheless should be that the ratio gets smaller as the wing is more elongated and larger as it gets wider, as we note in the text.

The main text (line 175) notes that "a larger D-V/A-P ratio in the wing disc corresponds to a rounder adult wing", and we have now added additional explanation to the Methods section where the measurement process is described ("Wing pouch shape analysis", line 749) that describes these intuitions about the relative values measured deriving from basic geometry.

3. I was concerned that the authors overstated that Hippo is irrelevant. The data show Hippo does contribute.
The authors revised the conclusion to "Hippo signaling contributes to, but does not fully explain, the influence of ds or fat on adult wing shape." They added wing size measurements (new Fig S3) and clarified allele strengths. The revised language is appropriately hedged. The addition of wts RNAi adult wings and hpo/wts RNAi wing discs in the new Fig S3 strengthens the ms. The key mechanistic distinction — that Hippo knockdown and Ds-Fat knockdown have opposite effects on myosin distribution (Fig S7) is a compelling new addition that supports their conclusion.

4. I was concerned that the 60 second distance measurement conflates tension with cell fluidity. Initial recoil velocity should have been measured.
The authors added recoil velocity measurements for wildtype and ds mutants (new Fig S6), showing consistency with circular ablation results. The new Fig S6 shows recoil velocity data confirming that proximal junctions have lower tension, and D-V boundary junctions have higher tension in ds mutants, corroborating the circular ablation inference. The axis of anisotropy (proximal vs D-V boundary) is now also explicitly stated throughout.

5. I pointed out that the axis along which cells were elongated was never specified.
The authors revised text and figures to clarify orientation of regions shown. The manuscript now clearly distinguishes proximal regions from D-V boundary regions, and the schematic in Figure 4A explicitly maps these locations on the wing disc.

6. I was concerned that low-magnification Sqh:GFP images were uninterpretable. High-resolution polarization analysis was needed.
The authors added high-resolution myosin images and quantitative assessments of myosin junctional polarity in control and ds mutants (revised Fig 5, including new panels I and J with rose plots). The polarity analysis showing loss of radial vs tangential myosin bias in ds mutants is a meaningful new result that goes beyond what was requested.
