# Supplementary material for: Dachsous-Fat signaling shapes the Drosophila wing through mechanical forces
Source: PLoS Biol. 2026 Jul 14;24(7):e3003883. doi: 10.1371/journal.pbio.3003883 (PMC13375139; doi:10.1371/journal.pbio.3003883)
Supplement: Attachment — Submitted filename: Response to Reviews_v6.docx [file pbio.3003883.s011.docx]

**Response to Reviewer’s Comments**

We thank all three reviewers for their thoughtful and detailed comments, which have helped us to improve the quality and clarity of our manuscript.

------------------------------------------------------------------------------ 
**Reviewer #1 (Evidence, reproducibility and clarity (Required)):** 
Summary: 
In this work, Tripathi et al address the open question of how the Fat/Ds pathway affects organ shape, using the Drosophila wing as a model. The Fat/Ds pathway is a conserved but complex pathway, interacting with Hippo signalling to affect growth and providing planar cell polarity that can influence cellular dynamics during morphogenesis. Here, authors use genetic perturbations combined with quantification of larval, pupal, and adult wing shape and laser ablation to conclude that the Ft/Ds pathway affects wing shape only during larval stages in a way that is at least partially independent of its interaction with Hippo and rather due to an effect on tissue tension and myosin II distribution. Overall the work is clearly written and well presented. I only have a couple major comments on the limitations of the work. 

Major comments: 
1. Authors conclude from data in Figures 1 and 2 that the Fat/Ds pathway only affects wing shape during larval stages. When looking at the pupal wing shape analysis in Figure 2L, however, it looks there is a difference in wt over time (6h-18h, consistent with literature), but that difference in time goes away in RNAi-ds, indicating that actually there is a role for Ds in changing shape during pupal stages, although the phenotype is clearly less dramatic than that of larval stages. No statistical test was done over time (within the genotype), however, so it's hard to say. I recommend the authors test over time - whether 6h and 18h are different in wild type and in ds mutant. I think this is especially important because there is proximal overgrowth in the Fat/Ds mutants, much of which is contained in the folds during larval stages. That first fold, however, becomes the proximal part of the pupal wing after eversion and contracts during pupal stages to elongate the blade (Aiguoy 2010, Etournay 2015). Also, according to Trinidad Curr Biol 2025, there is a role for Fat/Ds pathway in pupal stages. All of that to say that it seems likely that there would be a phenotype in pupal stages. It's true it doesn't show up in the adult wing in the experiments in Fig 1, but looking at the pupal wing itself is more direct - perhaps the very proximal effect is less prominent later, as there is potential for further development after 18hr before adulthood and the most proximal parts are likely anyway excluded in the analysis.

**Response**: Our main purpose in examining pupal wing shape was to emphasize that wings lacking ds are visibly abnormal even at early pupal stages. The reviewer makes the point that the change in shape from 6h to 18h APF is greater in control wings than in RNAi-ds wings. We have added quantitation of this to the revised manuscript as suggested. This difference could be interpreted as indicating that Ds-Fat signaling actively contributes to wing shape during pupal morphogenesis. However, given the genetic evidence that Ds-Fat signaling influences wing shape during larval growth, we favor the interpretation that it reflects consequences of Ds-Fat action during larval stages – eg, overgrowth of the wing, particularly the proximal wing and hinge as occurs in ds and fat mutants, could result in relatively less elongation during the pupal hinge contraction phase. We also now discuss this result and its interpretation in the revised manuscript.

2. I think there needs to be a mention and some discussion of the fact that the wing is not really flat. While it starts out very flat at 72h, by 96h and beyond, there is considerable curvature in the pouch that may affect measurements of different axis and cell shape. It is not actually specified in the methods, so I assume the measurements were taken using a 2D projection. Not clear whether the curvature of the pouch was taken into account, either for cell shape measurements presented in Fig 4 or for the wing pouch dimensional analysis shown in Fig 3, 6, and supplements. Do perturbations in Ft/Ds affect this curvature? Are they more or less curved in one or both axes? Such a change could affect the results and conclusions. The extent to which the fat/ds mutants fold properly is another important consideration that is not mentioned. For example, maybe the folds are deeper and contain more material in the ds/fat mutants, and that's why the pouch is a different shape? At the very least, this point about the 3D nature of the wing disc must be raised in discussion of the limitations of the study. For the cell shape analysis, you can do a correction based on the local curvature (calculated from the height map from the projection). For the measurement of A/P, D/V axes of the wing pouch, best would be to measure the geodesic distance in 3D, but this is not reasonable to suggest at this point. One can still try to estimate the pouch height/curvature, however, both in wild type and in fat/ds mutants. 

**Response**: The wing pouch measurements were done on 2D projections of wing discs that were already slightly flattened by coverslips, so there is not much curvature outside of the folds. We have revised the methods to make sure this is clear. While the reviewer is correct to point out that the absolute values measured can be affected by this, and by the folding of tissue in the hinge, our conclusions are based on the qualitative differences in proportions between genotypes and time points, and we wouldn’t expect these to differ significantly even if 3D distances were measured. Obtaining accurate 3D measures is technically more challenging and time consuming - it requires having spacers matching the thickness of the wing disc, which varies at different time points and genotypes, and then measuring distances in 3D across curved surfaces. What we have done to address this is to do a limited set of 3D measures on wild-type and *ds* mutant wing discs at early (72h AEL) and late (120h AEL) stages and provide images showing examples of what the measurements are based on that illustrate the curvature and folding (New Fig S2). The results show that the absolute values of the ratios measured are different, more so at 120h AEL, but the relative differences between wildtype and *ds* mutant are similar regardless of the measurement method (at 72h AEL *ds* mutants are 2.6/1.8 = 144% wider in 2D measures of the D-V/A-P ratio and 2.8/1.8 = 155% wider in 3D measures, and at 120h *ds* mutants are 2.6/1.7 = 153% wider in 2D measures and 1.3/0.9 = 144% wider in 3D measures). We think the similarity in relative differences validates our use of the 2D approximation, and we also now discuss the issue of wing disc curvature and folds in the text.

Minor comments: 
1. The analysis of the laser ablation is not really standard - usually one looks at recoil velocity or a more complicated analysis of the equilibrium shape using a model (e.g Shivakumar and Lenne 2016, Piscitello-Gomez 2023, Dye et al 2021). One may be able to extract more information from these experiments - nevertheless, I doubt the conclusions would change, given that that there seems to be a pretty clear difference between wt and ds (OPTIONAL).

**Response**: We have now also added measurements of recoil velocities in wildtype and ds mutants to complement our current analysis of circular cuts (Fig S6).

2. Figure 7G: I think you also need a statistical test between RNAi-ds and UAS-rokCA+RNAi-ds.

**Response**: We now include this statistical test in the revised manuscript (it shows that they are significantly different).

3. In the discussion, there is a statement: "However, as mutation or knock down of core PCP components, including pk or sple, does not affect wing shape... 59." Reference 59 is quite old and as far as I can tell shows neither images nor quantifications of the wing shape phenotype (not sure it uses "knockdown" either - unless you mean hypomorph?). A more recent publication Piscitello-Gomez et al Elife 2023 shows a very subtle but significant wing shape phenotype in core PCP mutants. It doesn't change your logic, but I would change the statement to be more accurate by saying "mutation of core PCP components has only subtle changes in adult wing shape"

**Response**: Thank-you for pointing this out, we have revised the manuscript accordingly and cited the Piscitello-Gomez et al paper.

**Referee cross-commenting** 

Reviewer2: 
Reviewer 2 makes the statement: "The distance along the AP boundary from the pouch border to DV midline is topologically comparable to the PD length of the adult wing. The distance along the DV boundary from A border to P border is topologically comparable to the AP length of the adult wing." 

I disagree - the DV boundary wraps around the entire margin of the adult wing (as correctly drawn with the pink line in Fig 2A). It is not the same as the wide axis of the adult wing (perpendicular to the AP boundary). It is not trivial to map the proximal-distal axis of the larval wing to the proximal-distal axis of the adult, due to the changes in shape that occur during eversion. Thus, I find it much easier to look at the exact measurement that the authors make, and it is much more standard in the field, rather than what the reviewer suggests. Alternatively, one could I guess measure in the adult the ratio of the DV margin length (almost the circumference of the blade?) to the AP boundary length. That may be a more direct comparison. 
Actually the authors leave out the term "boundary" - what they call AP is actually the AP boundary, not the AP axis, and likewise for the DV - what they measure is DV boundary, but I only noticed that in the second read-through now. Just another note, these measurements of the pouch really only correspond to the very distal part of the wing blade, as so much of the proximal blade comes from the folds in the wing disc. Therefore, a measurement of only distal wing shape would be more comparable.

**Response**: We thank Reviewer 1 for their comments here. In terms of the region measured, we measure to the inner Wg ring in the disc, the location of this ring in the adult is actually more proximal than described above (eg see Fig 1B of Liu, X., Grammont, M. & Irvine, K. D. Roles for scalloped and vestigial in regulating cell affinity and interactions between the wing blade and the wing hinge. *Developmental Biology* **228**, 287–303 (2000)), and this defines roughly the region we have measured in adult wings.

Reviewer 2 states that authors cannot definitively conclude anything about mechanical tension from their reported cutting data because the authors have not looked at initial recoil velocity. 
**I strongly disagree.** The wing disc tissue is elastic on much longer timescales than what's considered after laser ablation (even hours), and **the shape of the tissue after it equilibrates from a circular cut (1-2min) can indeed be used to infer tissue stresses (see Dye et al Elife 2021, Piscitello-Gomez et al eLife 2023, Tahaei et al arXiv 2024).** In the wing disc, the direction of stresses inferred from initial recoil velocity are correlated with the direction of stresses inferred from analysing the equilibrium shape after a circular cut. Rearrangements, a primary mechanism of fluidization in epithelia, does not occur within 1'. Analysing the equilibrium shape after circular ablation may be more accurate for assessing tissue stresses than initial recoil velocity - in Piscitello-Gomez et al 2023, the authors found that a prickle mutation (PCP pathway) affected initial recoil velocity but not tissue stresses in the pupal wing. Such equilibrium circular cuts have also been used to analyze stresses in the avian embryo, where it correlates with directions of stress gathered from force inference methods (Kong et al Scientific Reports 2019). The Tribolium example noted by the reviewer is on the timescale of tens to hundreds of minutes - much longer than the timescale of laser ablation retraction. It is true the analysis of the ablation presented in this paper is not at the same level as those other cited papers and could be improved. But I don't think the analysis would be improved by additional experiments doing timelapse of initial retraction velocity.

**Response**: We thank Reviewer 1 for their comments here, and we are keeping the circular ablation data in the manuscript. We are also adding analysis of retraction velocities after cutting individual junctions in wildtype and ds mutant wing discs.

Reviewer 2 states "If cell anistropy is caused by polarized myosin activity, that activity is typically polarized along the short edges not long edges" 
Not true in this case. Myosin II accumulates along long boundaries (Legoff and Lecuit 2013). "Therefore, interpreting what causes the cell anistropy and how DS regulates it is difficult," Agreed - but this is well beyond the scope of this manuscript. The authors clearly show that there is a change of cell shape, at least in these two regions. Better would be to quantify it throughout the pouch and across multiple discs. Similar point for myosin quantifications - yes, polarity would be interesting and possible to look at in these data, and it would be better to do so on multiple discs, but the lack of overall myosin on the junctions shown here is not nothing. Interpreting what Ft/Ds does to influence tension and myosin and eventually tissue shape is a big question that's not answered here. I think the authors do not claim to fully understand this though, and maybe further toning down the language of the conclusions could help.

**Response**: We greatly appreciate the comments of Reviewer 1 here, and we have also now added quantitation of myosin across multiple discs and included examples of higher magnification myosin images and assessments of polarity for wildtype and *ds* mutants.

Reviewer 3: 
I agree with many of the points raised by Reviewer 3, in particular that relevant for Fig 1. The additional experiments looking at myosin II localization and laser ablation in the other perturbations (Hippo and Rok mutants/RNAi) would certainly strengthen the conclusions. 

**Response**: The Reviewer 3 comment on Fig 1 requests Ab stains to assess recovery of expression after downshift, which we now include (New Fig S1).

Doing laser cutting in all the genotypes examined is beyond the scope of what we consider reasonable for the manuscript, but we have now added additional examination of myosin levels in additional genotypes and in combinations of *ds* RNAi with manipulations of *rok.* The effects of Rok manipulations on myosin levels and on recoil velocity in wing discs have also been described previously (eg see Rauskolb et al 2014, DOI: 10.1016/j.cell.2014.05.035).

Reviewer #1 (Significance (Required)): 
I think the work provides a clear conceptual advance, arguing that the Ft/Ds pathway can influence mechanical stress independently of its interaction with Hippo and growth. Such a finding, if conserved, could be quite important for those studying morphogenesis and Fat function in this and other organisms. For this point, the genetic approach is a clear strength. Previous work in the Drosophila wing has already shown an adult wing phenotype for Ft/Ds mutations that was attributed to its role in the larval growth phase, as marked clones show aberrant growth in mutants. The novelty of this work is the dissection of the temporal progression of this phenotype and how it relates to Hippo and myosin II activation. It remains unclear exactly how Ft/Ds may affect tissue tension, except that it involves a downregulation of myosin II - the mechanism of that is not addressed here and would involve considerable more work. I think the temporal analysis of the wing pouch shape was quite revealing, providing novel information about how the phenotype evolves in time, in particular that there is already a phenotype quite early in development. As mentioned above, however, the lack of consideration of the wing disc as a 3D object is a potential limitation. While the audience is likely mostly developmental biologists working in basic research, it may also interest those studying the pathway in other contexts, including in vertebrates given its conservation and role in other processes. 


**Reviewer #2 (Evidence, reproducibility and clarity (Required)):** 
The manuscript begins with very nice data from a ts sensitive period experiment. Instead of a ts mutation, the authors induced RNAi in a temperature dependent manner. The results are striking and strong. Knockdown of FT or DS during larval stages to late L3 changed shape while knockdown of FT or DS during later pupal stages did not. This indicates they are required during larval, not pupal stages of wing development for this shape effect. They did shift-up or shift-down at "early pupa stage" but precisely what stage that means was not described anywhere in the manuscript. White prepupal? Time? Likewise a shift-down was done at "late L3" but that meaning is also vague. Moreover, I was surprised to see they did not do a shift-up at the late L3 stage, to give completeness to the experiment. Why?

**Response**: We have now added more precise descriptions of the timing and added the requested late L3 shift-up experiment.

Looking at the "shape" of the larval wing pouch they see a difference in the mutants. The pouch can be approximated as an ellipse, but with differing topology to the adult wing. Here, they muddled the analysis. The adult wing surface is analogous to one hemisphere of the larval wing pouch, ie., either dorsal or ventral compartment. The distance along the AP boundary from the pouch border to DV midline is topologically comparable to the PD length of the adult wing. The distance along the DV boundary from A border to P border is topologically comparable to the AP length of the adult wing. They confusingly call this latter metric the "DV length" and the former metric the "AP length" , and in fact they do not measure the PD length but PD+DP length. Confusing. Please change to make this consistent with earlier analysis of the adult and invert the reported ratio and divide by two.

Then you would find the larval PD/AP ratio is smaller in the FT and DS mutants than wildtype, which resembles the smaller PD/AP ratio seen in the mutant adult wings. Totally consistent and also provides further evidence with the ts experiments that FT and DS exert shape effects in the larval phase of life. 

**Response**: As noted by Reviewer 1 in cross-referencing, some of the statements made by Reviewer 2 here are incorrect, eg “The distance along the DV boundary from A border to P border is topologically comparable to the AP length of the adult wing,” as it's actually the circumference. They are correct where they note that the A-P length we measure in the discs is actually equivalent to 2x the adult wing length, since we are measuring along both the dorsal and ventral wing, but this makes no difference to the analysis as the point is to compare shape between time points and genotypes, not to make inferences based on the absolute numbers obtained. The numerical manipulations suggested are feasible but we think they are unnecessary.

The remainder of the manuscript has experimental results that are more problematic, and really the authors do not figure out how the shape effect in larval stages is altered. I outline below the main problems. 

1. They compare the FT DS shape phenotypes to those of mutants or knockdowns in Hippo pathway genes (Hippo is known to be downstream of FT and DS). They find these Hippo perturbations do have shape effects trending in same direction as FT and DS effects. Knockdown reduces the PD/AP ratio while overexpressing WARTS increases the PD/AP ratio. The effect magnitudes are not as strong, but then again, they are using hypomorphic alleles and RNAi, which often induces partial or hypomorphic phenotypes. The effect strength is comparable when wing pouches are young but then dissipates over time, while FT and DS effects do not dissipate over time. The complexity of the data do not negate the idea that Hippo signaling is also playing some role and could be downstream of FT and DS in all of this. But the authors really downplay the data to the point of stating "These results imply that Ds-Fat influences wing pouch shape during wing disc growth separately from its effects on Hippo signaling." I think a more expansive perspective is needed given the caveats of the experiments.

**Response**: Our results emphasize that the effects of Ds-Fat on wing shape cannot be explained solely by effects on Hippo signaling, eg as we stated on page 7 “*These observations suggest that Hippo signaling contributes to, but does not fully explain, the influence of ds or fat on adult wing shape.*” We also note that impairment of Hippo signaling has similar effects in younger discs, but very different effects in older discs, which clearly indicates that they are having very different effects during disc growth; we have revised the text to make sure our conclusions are clearer.

The reviewer wonders whether some of the differences could be due to the nature of the alleles or gene knockdown. First, the *ex*, *ds*, and *fat* alleles that we use are null alleles (eg see FlyBase), so it is not correct to say that we use only hypomorphic alleles and RNAi. We do use a hypomorphic allele for wts, and RNAi for hpo, because null alleles in these genes are lethal so adult wings could not be examined. A further issue that is not commented on by the reviewer, but is relevant here, is that there are multiple inputs into Hippo signaling, so of course even a null allele for *ex*, *ds* or *fat* is not a complete shutdown of Hippo signaling. Nonetheless, one can estimate the relative impairment of Hippo signaling by measuring the increased size of the wings, and from this perspective the knockdown conditions that we use are associated with roughly comparable levels of Hippo pathway impairment, so we stand by our results. We do however, recognize that this could be discussed more clearly in the text, and have tried to do so, and we have also now added adult wing size measurements for various genotypes examined to the revised manuscript (New Fig S3).

Puzzlingly, this lack of taking seriously a set of complex results does not transfer to another set of experiments in which they inhibit or activate ROK, the rho kinase. When ROK is perturbed, they also see weak effects on shape when compared to FT or DS perturbation. This weakness is seen in adults, larvae, clones and in epistasis experiments. The epistasis experiment in particular convincingly shows that constitutuve ROK activation is not epistatic to loss of DS; in fact if anything the DS phenotype suppresses the ROK phenotype. These results also show that one cannot simply explain what FT and DS are doing with some single pathway or effector molecule like ROK. It is more complex than that.

What I really think was needed were experiments combining FT and DS knockdown with other mutants or knockdowns in the Hippo and Rho pathways, and even combining Hippo and Rho pathway mutants with FT or DS intact, to see if there are genetic interactions (additive, synergistic, epistatic) that could untangle the phenotypic complexity.

**Response**: We’re not sure we understand these comments. We do not claim that what Fat or Ds do could be explained simply by manipulation of Rok. Examination of wings and wing discs where *ds* is combined with Rho manipulations were in Fig 7, and Hippo and Rho pathway manipulation combinations were in Fig S5 (now Fig S7). We've now also added some additional examination of myosin levels in these genotypes. We don’t think that combining *ds* or *fat* mutations with other Hippo pathway mutations would be informative, as it is well established that Ds-Fat are upstream regulators of Hippo signaling. We have, however, now added examination of wings in which manipulations of Dachs are combined with manipulations of Rok.

2. Laser cutting experiments were done to see if there is anisotropy in tissue tension within the wing pouch. This was to test a favored idea that FT and DS activity generates anisotropy in tissue tension, thereby controlling overall anisotropic shape of the pouch. However there is a fundamental flaw to their laser cutting analysis. Laser cutting is a technique used to measure mechanical tension, with initial recoil velocity directly proportional to the tissue's tension. By cutting a small line and observing how quickly the edges of the cut snap apart, people can quantify the initial recoil velocity and infer the stored mechanical stress in the tissue at the time of ablation. Live imaging with high-speed microscopy is required to capture the immediate response of the tissue to the cut since initial recoil velocity occurs in the first few seconds. A kymograph is created by plotting the movement of the tissue edges over this time scale, perpendicular to the cut. The initial recoil velocity is the slope of the kymograph at time zero, representing how fast the severed edges move apart. A higher recoil velocity indicates higher mechanical tension in the tissue. However, the authors did not measure this initial recoil velocity but instead measured the distance between the severed edges at one time point: 60 seconds after cutting. This is much later than the time point at which the recoil usually begins to dissipate or decay. This decay phase typically lasts a minute or two, during which time the edges continue to separate but at a progressively slower rate. This time-dependent decay of the recoil reveals whether the tissue behaves more like a viscous fluid or an elastic solid. Therefore, the distance metric at 60 seconds is a measurement of both tension and the material properties of the cells. One cannot know then whether a difference in the distance is due to a difference in tension or fluidity of the cells. If the authors made measurements of edge separation at several time points in the first 10 seconds after ablation, they can deconvolute the two. Otherwise their analysis is inconclusive. Anisotropy in recoil could be caused by greater tissue fluidity along one axis. Observing a gradient of cell fluidity in a tissue along one axis of a tissue has been observed in the amnioserosa of Tribolium for example. (Related and important point - was the anisotropy of recoil oriented along the PD or AP axis or not oriented to either axis, this key point was never stated).. 

The authors cannot definitiviely conclude anything about mechanical tension from their reported cutting data. 

**Response**: As noted by Reviewer 1 in cross-commenting, there is no fluidity on a time scale of 1 minute in the wing disc, and circular ablations are an established methods to investigate tissue stress. We choose the circular ablation method in part because it interrogates stress over a larger area, whereas cutting individual junctions is subject to more variability, particularly as the orientation of the junction (eg radial vs tangential) impacts the tension detected in the wing disc. Nonetheless, to address the Reviewer’s concern here we have now added recoil velocity measurements to the revised manuscript for wildtype and ds mutants (New Fig S6); the results obtained are consistent with the circular ablation and myosin intensity measures.

3. They measured the eccentricity of wing pouch cells near the pouch border, and found they were highly anisotropic compared to DS mutant cells at comparable locations. Cells were elongated but again what if either axis (PD or AP) they were elongated along was never stated. If cell anistropy is caused by polarized myosin activity, that activity is typically polarized along the short edges not long edges. Thus, recoil velocity after laser cutting would be stonger along the axis aligned with short cell edges. It looks like the cutting anisotropy they see is greater along the axis aligned with long cell edges. Of course, if the cell anisotropy is caused by a pulling force exerted by the pouch boundary, then it would stretch the cells. This would in fact fit their cutting data. But then again, the observed cell anisotropy could also be caused by variation in the fluid-solid properties of the wing cells as discussed earlier. Compression of the cells then would deform them anisotropically and produce the anisotropic shapes that were observed, Therefore, interpreting what causes the cell anistropy and how DS regulates it is difficult,

**Response**: As noted by Reviewer 1 in cross-commenting, it is well established that tension and myosin are higher along long edges in the proximal wing, which is what we show here in the wildtype control. However, we acknowledge that we could do a better job of explaining this and making the orientation of the regions shown in these experiments clearer and, we have revised the text and figures to do this.

4. The imaging and analysis of the myosin RLC by GFP tagging is also flawed. SQH-GFP is a tried and true proxy for myosin activity in Drosophila. Although the authors image the wing pouch of wildtype and DS mutants. they did so under low magnification to image the entire pouch. This gives a "low-res" perspective of overall myosin but what they needed to do was image at high magnification in that proximal region of the pouch and see if Sqh-GFP is polarized in wildtype cells along certain cell edges aligned with an axis. And if such a polarity is observed, is it present or absent in the DS mutant. From the data shown in Figure 5, I cannot see any significant difference between wildtype and knocked down samples at this low resolution. Any difference, if there is any, is not really interpretable. 

**Response**: We agree that examination of myosin localization at high resolution to see if it is polarized is a worthwhile experiment. We did in fact do this, and myosin (Sqh:GFP) appeared unpolarized in *ds* mutants. However, due to the lower levels of myosin in *ds* mutants we didn’t feel confident in our qualitative assessments, so we didn’t include it. We now recognize that this was a mistake, and we now include examples high resolution myosin images, and also quantitative assessments of myosin localization, in control and in *ds* mutants in the revised manuscript (Fig. 5). The results show that there is a loss of the polarization of myosin localization that is normally evident in proximal wing disc cells.

In conclusion, the manuscript has multiple problems that make it imposiible for the authors to make the claims they make in the current manuscript. And even if they calibrated their interpretations to fit the data, there is not much of a simple clear picture as to how FT and DS regulate pouch eccentricity in the larval wing. 

Reviewer #2 (Significance (Required)): 
This manuscript describes experiments studying the role that the protocadherins FAT and DACHSOUS play in determining the two dimensional "shape" of the fruit fly wing. By "shape", the manuscript really means how much the wing's outline, when approximated as an ellipse, deviates from a circle. The elliptical approximations of FT and DS mutant wings more closely resemble a circle compared to the more eccentric wildtype wings. This suggests the molecules contribute to anisotropic growth in some way. A great deal of attention has been paid on how FT and DS regulate overall organ growth and planar cell polarity, and the Irvine lab has made extensive contributions to these questions over the years. Somewhat understudied is how FT and DS regulate wing shape, and this manuscript focuses on that. It follows up on an interesting result that the Irvine lab published in 2019, in which mud mutants randomized spindle pole orientation in wing cells but did not change the eccentricity of wings, ruling out biased cell division orientation as a mechanism for the anisotropic growth. 


**Reviewer #3 (Evidence, reproducibility and clarity (Required)):** 
Summary 
The authors investigate the mechanisms underlying epithelial morphogenesis using the Drosophila wing as a model system. Specifically, they analyze the contribution of the conserved Fat/Ds pathway to wing shape regulation. The main claim of the manuscript is that Ds/Fat controls wing shape by regulating tissue mechanical stress through MyoII levels, independently of Hippo signaling and tissue growth. 

Major Comments 
To support their main conclusions, the authors should address the following major points and consider additional experiments where indicated. Most of the suggested experiments are feasible within a reasonable timeframe, while a few are more technically demanding but would substantially strengthen the manuscript's central claims. 

Figure 1: 
The authors use temperature-sensitive inactivation of Fat or Ds to determine the developmental window during which these proteins regulate wing shape. To support this claim, it is essential to demonstrate that upon downshift during early pupal stages, Ds or Fat protein levels are restored to normal. For consistency, please include statistical analyses in Figure 1P and ensure that all y-axis values in shape quantifications start at 1. 

**Response**: We have now included the requested antibody stains for Fat (Ds antibody is unfortunately no longer readily available, but the point made by the reviewer can be addressed by Fat as the approach and results are the same for both genes). What we found is that Fat staining was recovered within 24h after downshift, but not within 6h after downshift (and only partially at 12h). As our late L3 downshift is done at ~8-12h before puparium formation, we also now include an additional experiment in which the upshift was done at mid third instar (24h before puparium formation), which results in wings that are almost, but not quite, as round as observed with continuous shift. We think this is because of Fat recovery during larval stages in this experiment but acknowledge that we can't completely rule out effects in pupae from this experiment due to the lag in recovery of Fat protein (although we think this is unlikely because upshift experiments also implicate larval development as the time of Ds-Fat action). We have consequently modified the text to be more cautious in interpreting these experiments. We have also added the requested statistical analysis to Fig 1, and adjusted the scales as requested.

Figure 2: 
The authors propose that wing shape is regulated by Fat/Ds during larval development. However, Figure 2L suggests that wing elongation occurs in control conditions between 6 and 12 h APF, while this elongation is not observed upon Ds RNAi. The authors should therefore perform downshift experiments while monitoring wing shape during the pupal stage to substantiate their main claim. In addition, equivalent data for Fat loss of function should be included to support the assertion that Fat and Ds act similarly. 

**Response**: As noted in our response to point 1 of Reviewer 1, we agree that there is relatively more elongation in control wings than in ds RNAi wings, but we think this likely reflects effects of ds on growth during larval stages, and we have revised the manuscript to comment on this. We have also added the suggested examination of fat RNAi pupal wings.

The suggested examination of pupal wing shape in downshift experiments is unfortunately not feasible. In our preliminary response to Reviewers, we noted that our temperature shift experiments expressing *ds* or *fat* RNAi are done using the UAS-Gal4-Gal80^ts^ system. We also use the UAS-Gal4 system to mark the pupal wing. If we do a downshift experiment, then expression of the fluorescent marker will be shut down in parallel with the shutdown of ds or fat RNAi, so the pupal wings would no longer be visible. We note that the academic editor then suggested that the experiment could be done in a different way, using a GAL80-insensitive rotund-LexA driver that could in principle be used to drive a marker independently of the nubbin-Gal4 UAS-ds-RNAi used. We obtained flies with the transgenes needed for this experiment, and created the necessary fly lines, but then found that expression of the marker we used (LexAop2-IVS-myr::GFP) under control of rotund-LexA is temperature sensitive, as we could detect the marker in pupal wings in vivo at 29°C, but not at 18°C, so this approach also proved unfeasible.

Figure 3: 
The authors state that "These observations indicate that Ds-Fat signaling influences wing shape during the initial formation of the wing pouch, in addition to its effects during wing growth." This conclusion is not fully supported, as the authors only examine wing shape at 72 h AEL. At this stage, fat or ds mutant wings already display altered morphology. The authors could only make this claim if earlier time points were fully analyzed. In fact, the current data rather suggest that Ds function is required before 72 h AEL, as a rescue of wing shape is observed between 72 and 120 h AEL.

**Response**: First, I think we are largely in agreement with the Reviewer, as the basis for our saying that DS-Fat are likely required during initial formation of the wing pouch is that our data show they must be required before 72 h AEL, as we already see a phenotype at this time. Second, 72 h is the earliest that we can look using Wg expression as a marker, as at earlier stages (ie 64h AEL) it is in a ventral wedge rather than a ring around the future wing pouch + DV line (eg see Fig 8 of Tripathi, B. K. & Irvine, K. D. The wing imaginal disc. Genetics (2022) doi:10.1093/genetics/iyac020.). We have revised the text to try to clarify the interpretation here.

Figure 4: 
The authors state that "The influence of Ds-Fat on wing shape is not explained by Hippo signaling." However, this conclusion is not supported by their data, which show that partial loss of ex or hippo causes clear defects in wing shape. In addition, the initial wing shape is affected in wts and ex mutants, and hypomorphic alleles were used for these experiments. Therefore, the main conclusion requires revision. It would be useful to include a complete dataset for hippo RNAi, ex, and wts conditions in Figure S1. 
The purpose and interpretation of the InR^CA experiments are also unclear. While InR^CA expression can increase tissue growth, Hippo signaling has functions beyond growth control. Whether Hippo regulates tissue shape through InR^CA-dependent mechanisms remains to be clarified. 

**Response**: As noted in our response to point 1 of Reviewer 2 - our results emphasize that the effects of Ds-Fat on wing shape cannot be explained solely by effects on Hippo signaling, eg as we had stated on page 7 “*These observations suggest that Hippo signaling contributes to, but does not fully explain, the influence of ds or fat on adult wing shape.*” We also note that impairment of Hippo signaling has similar effects in younger discs, but very different effects in older discs, which clearly indicates that they are having very different effects during disc growth. We have revised the text to try to clarify our conclusions.

While we used a hypomorphic allele for *wts*, because null alleles are lethal, the ex allele that we used is described in Flybase as an amorph, not a hypomorph, and as noted in our response to Reviewer 2, we have also added some discussion about the relative strength of effects on Hippo signaling in different genotypes.

In Fig S1, we had shown adult wings for ex[e1] and RNAi-Hpo, and wing discs for wts[P2]/wts[x1], and for ex[e1]. The *wts* mutant combination does not survive to adult so we can’t include this. We have however, now added *wts* RNAi adult wings, and hpo RNAi and wts RNAi wing discs (New Fig S3).

The purpose of including InR^CA experiments is to try to separate effects of Hippo signaling from effects of growth, because InR signaling manipulation provides a distinct mechanism for increasing growth. We have revised the text to try to make this clearer.

Figure 5: 
This figure presents images of MyoII distribution, but no quantification across multiple samples is provided. Moreover, the relationship between changes in tissue stress and MyoII levels remains unclear. Performing laser ablation and MyoII quantification on the same samples would provide stronger support for the proposed conclusions.

**Response**: We have now added quantitation of averages of myosin intensity in distal and proximal regions across multiple discs for control and ds RNAi discs (The RNAi experiments were used so that myosin levels in anterior regions could be used as an internal control for comparison to the effects in posterior cells, where the RNAi is expressed); the results obtained are consistent with the earlier descriptions.

Both the myosin imaging and the laser ablation were done on the same genotypes (*wildtype* and *ds*) at the same ages (108 h AEL) so we think it is valid to directly compare them. The imaging conditions for laser ablation and myo quantification are quite different however, so it’s not feasible to do them at the same time on the same disc (For laser ablations we do a single Z plane and a single channel (has to include Ecad, or an equivalent junctional marker) on live discs, so that fast imaging can be done. For Myo imaging we do multiple Z stacks and multiple channels (eg Ecad and Myo), which is not compatible with the fast imaging needed for analysis of laser ablations).

Figure 6: 
It is unclear when Rok RNAi and Rok^CA misexpression were induced. To substantiate their claims, the authors should measure both MyoII levels and mechanical tension under the different experimental conditions in which wing shape was modified through Rok modulation (i.e. the condition shown in Fig. 7G). For comparison, fat and ds data should be added to Fig 6H. 
Overall, the effects of Rok modulation appear milder than those of Fat manipulation. Given that Dachs has been shown to regulate tension downstream of Fat/Ds, it would be informative to determine whether tissue tension is altered in dachs mutant wings and to assess the relative contribution of Dachs- versus MyoII-mediated tension to wing shape control. It would also be interesting to test whether Rok activation can rescue dachs loss-of-function phenotypes.

**Response**: In these Rok experiments there was no separate temporal control of Rok RNAi or Rok^CA expression, they were expressed under nub-Gal4 control throughout development.

To try to address the other issues raised here, we have added examination of myosin in combinations of *ds* RNAi and *rok* manipulation to the revised manuscript (Fig 7). Data for *fat* and *ds* comparable to that shown in Fig 6H (wing pouch shape) is already presented in Fig 3D and we don’t think it's necessary to show it again in in this Figure. We agree that the effects of Rok manipulations are milder than those of Fat manipulations; as we try to discuss, this could be because the pattern or polarity of myosin is also important, not just the absolute level. We have also now added assessment of myosin polarity in control and *ds* mutant wing discs. The suggestion to also look at *dachs* mutants is logical, but it is extremely difficult to get *dachs* null mutant adult flies, and available *dachs* RNAi flies have off-target effects, so we used UAS-ft as a proxy for loss of Dachs to add the requested experiments looking at adult wing shape +/- rok manipulations in the absence of Dachs. In addition, we added an "activated" Dachs (a Zyxin:Dachs fusion protein previously described in Pan et al 2013) that provides further evidence that the effects of Ds-Fat are mediated through Dachs, and looked at wing shape in this genotype +/- rok, and we also included myosin staining in *dachs* mutants and in Zyx:Dachs-expressing and UAS-ft-expressing wing discs (New Fig 8). Due to the high level of time and effort involved we have not done laser cutting for all these genotypes, but we have quantified myosin, which is a reasonable proxy for tension, and laser cutting for rok RNAi and rok.CA have been reported previously.

Figure 7: 
The authors use genetic interactions to support their claim that Fat controls wing shape independently of Hippo signaling. However, these interactions do not formally exclude a role for Hippo. Moreover, previous work has shown that tissue tension regulates Hippo pathway activity, implying that any manipulation of tension could indirectly affect Hippo and growth. To provide more direct evidence, the authors should further analyze MyoII localization and tissue tension under the various experimental conditions tested (as also suggested above). 

**Response**: As discussed above, our data clearly show that Fat has effects independently of Hippo signaling that are crucial for its effects on wing shape, but we did not mean to imply that the regulation of Hippo signaling by Fat makes no contribution to wing shape control, and we have revised the text to try to make this clearer. We have also added analysis of Myosin localization in some additional genotypes. The most obvious difference in regards to Fat vs Hippo effects is that knockdown of Ds-Fat signaling (*ds* mutants, *ds* RNAi, Zyx:Dachs fusion) leads to lower myosin in the proximal wing disc, whereas knockdown of Hippo signaling (UAS-hpo, ex or wts mts) leads to similar or even higher higher myosin in the proximal wing disc (New Fig S7).

Reviewer #3 (Significance (Required)): 
How organ growth and shape are controlled remains a fundamental question in developmental biology, with major implications for our understanding of disease mechanisms. The Drosophila wing has long served as a powerful and informative model to study tissue growth and morphogenesis. Work in this system has been instrumental in delineating the conserved molecular and mechanical processes that coordinate epithelial dynamics during development. The molecular regulators investigated by the authors are highly conserved, suggesting that the findings reported here are likely to be of broad biological relevance. 

Previous studies have proposed that anisotropic tissue growth regulates wing shape during larval development and that such anisotropy induces mechanical responses that promote MyoII localization (Legoff et al., 2013, PMID: 24046320; Mao et al., 2013, PMID: 24022370). The Ds/Fat system has also been shown to regulate tissue tension through the Dachs myosin, a known modulator of the Hippo/YAP signaling pathway. As correctly emphasized by the authors, the respective contributions of anisotropic growth and mechanical tension to wing shape control remain only partially understood. The current study aims to clarify this issue by analyzing the role of Fat/Ds in controlling MyoII localization and, consequently, wing shape. This represents a potentially valuable contribution. However, the proposed mechanistic link between Fat/Ds and MyoII localization remains insufficiently explored. Moreover, the role of MyoII is not fully discussed in the broader context of Dachs function and its known interactions with MyoII (Mao et al., 2011, PMID: 21245166; Bosveld et al., 2012, PMID: 22499807; Trinidad et al., 2024, PMID: 39708794). Most importantly, the experimental evidence supporting the authors' conclusions would benefit from further strengthening. It should also be noted that disentangling the relative contributions of anisotropic growth and MyoII polarization to tissue shape and size remains challenging, as MyoII levels are known to increase in response to anisotropic growth (Legoff et al., 2013; Mao et al., 2013), and mechanical tension itself can modulate Hippo/YAP signaling (Rauskolb et al., 2014, PMID: 24995985).
