# Supplementary material for: Dachsous-Fat signaling shapes the Drosophila wing through mechanical forces
Source: PLoS Biol. 2026 Jul 14;24(7):e3003883. doi: 10.1371/journal.pbio.3003883 (PMC13375139; doi:10.1371/journal.pbio.3003883)
Supplement: Peer review history

# Author comment: Author response to Decision Letter 0

**DOI:** 10.1371/journal.pbio.3003883.r001

10 Nov 2025

Attachment Submitted filename: 160578_1_rebuttal_3571377_t5dx92.docx

# Editor report: Decision Letter 0

**DOI:** 10.1371/journal.pbio.3003883.r002

**Authors:** Ines Alvarez-Garcia

21 Nov 2025

Dear Dr Irvine,

Thank you for submitting your manuscript via Review Commons entitled "Dachsous-Fat Signaling Shapes the Drosophila Wing through Mechanical Forces" for consideration as a Research Article by PLOS Biology.

Your manuscript, associated reviews and revision plan have now been evaluated by the PLOS Biology editorial staff as well as by an academic editor with relevant expertise, and I am writing to let you know that we would like to invite you to submit a revision.

However, before we can send you the decision, we need you to complete your submission by providing the metadata that is required for full assessment. To this end, please login to Editorial Manager where you will find the paper in the 'Submissions Needing Revisions' folder on your homepage. Please click 'Revise Submission' from the Action Links and complete all additional questions in the submission questionnaire.

Once your full submission is complete, your paper will undergo a series of checks. After your manuscript has passed the checks we will send you the decision with feedback on your revision plan. To provide the metadata for your submission, please Login to Editorial Manager (https://www.editorialmanager.com/pbiology) within two working days, i.e. by Nov 25 2025 11:59PM.

During the process of completing your manuscript submission, you will be invited to opt-in to posting your pre-review manuscript as a bioRxiv preprint. Visit http://journals.plos.org/plosbiology/s/preprints for full details. If you consent to posting your current manuscript as a preprint, please upload a single Preprint PDF.

Feel free to email us at plosbiology@plos.org if you have any queries relating to your submission.

Kind regards,

Ines

--

Ines Alvarez-Garcia, PhD

Senior Editor

PLOS Biology

ialvarez-garcia@plos.org

# Author comment: Author response to Decision Letter 1

**DOI:** 10.1371/journal.pbio.3003883.r003

21 Nov 2025

Attachment Submitted filename: 160578_1_rebuttal_3571377_t5dx92_auresp_1.docx

# Editor report: Decision Letter 1

**DOI:** 10.1371/journal.pbio.3003883.r004

**Authors:** Ines Alvarez-Garcia, Ines Alvarez-Garcia

28 Nov 2025

Dear Dr Irvine,

Thanks again for submitting via Review Commons your manuscript entitled "Dachsous-Fat Signaling Shapes the Drosophila Wing through Mechanical Forces". The manuscript, associated reviews and proposed revision plan have been evaluated by the PLOS Biology editors and an Academic Editor with relevant expertise.

The previous reviews are attached below for your reference. After discussing the revision plan with the Academic Editor, we are mostly satisfied with the experiments proposed with one exception. It is mentioned that the downshift experiments suggested by Reviewer 3 to monitor wing shape regulation by Ft/Ds during the pupal stages are not feasible. However, there is a recent publication from the Vincent lab (PMID: 37769663) that describes a GAL80-insensitive rotund-LexA driver that could in principle be used to drive a marker like CD8::RFP independently of the nubbin>ds-RNAi used. We think that the experiment does address an important point of the paper and should be performed if possible.

Given the extent of revision needed, we cannot make a decision about publication until we have seen the revised manuscript and your response to the reviewers' comments. Your revised manuscript is likely to be sent for further evaluation by all or a subset of the reviewers.

In addition to these revisions, you will need to complete some formatting changes, which you will receive in a follow up email. A member of our team will be in touch with a set of requests shortly.

We expect to receive your revised manuscript within 3 months. Please email us (plosbiology@plos.org) if you have any questions or concerns, or would like to request an extension.

At this stage, your manuscript remains formally under active consideration at our journal; please notify us by email if you do not intend to submit a revision so that we may withdraw it.

**IMPORTANT - SUBMITTING YOUR REVISION**

Your revisions should address the specific points made by each reviewer. Please submit the following files along with your revised manuscript:

1. A 'Response to Reviewers' file - this should detail your responses to the editorial requests, present a point-by-point response to all of the reviewers' comments, and indicate the changes made to the manuscript.

*NOTE: In your point-by-point response to the reviewers, please provide the full context of each review. Do not selectively quote paragraphs or sentences to reply to. The entire set of reviewer comments should be present in full and each specific point should be responded to individually, point by point.

You should also cite any additional relevant literature that has been published since the original submission and mention any additional citations in your response.

2. In addition to a clean copy of the manuscript, please also upload a 'track-changes' version of your manuscript that specifies the edits made. This should be uploaded as a "Revised Article with Changes Highlighted" file type.

3. Resubmission Checklist

When you are ready to resubmit your revised manuscript, please refer to this resubmission checklist: https://plos.io/Biology_Checklist

To submit a revised version of your manuscript, please go to https://www.editorialmanager.com/pbiology/ and log in as an Author. Click the link labelled 'Submissions Needing Revision' where you will find your submission record.

Please make sure to read the following important policies and guidelines while preparing your revision and fulfil the editorial requests:

a) *PLOS Data Policy*

Please note that as a condition of publication PLOS' data policy (http://journals.plos.org/plosbiology/s/data-availability) requires that you make available all data used to draw the conclusions arrived at in your manuscript. If you have not already done so, you must include any data used in your manuscript either in appropriate repositories, within the body of the manuscript, or as supporting information (N.B. this includes any numerical values that were used to generate graphs, histograms etc.). Please also indicate in each figure legend where the data can be found. For an example see here: http://www.plosbiology.org/article/info%3Adoi%2F10.1371%2Fjournal.pbio.1001908#s5

b) *Published Peer Review*

Please note while forming your response, if your article is accepted, you may have the opportunity to make the peer review history publicly available. The record will include editor decision letters (with reviews) and your responses to reviewer comments. If eligible, we will contact you to opt in or out. Please see here for more details:

https://blogs.plos.org/plos/2019/05/plos-journals-now-open-for-published-peer-review/

c) *Blot and Gel Data Policy*

Please provide the original, uncropped and minimally adjusted images supporting all blot and gel results reported in an article's figures or Supporting Information files. We will require these files before a manuscript can be accepted so please prepare them now, if you have not already uploaded them. Please carefully read our guidelines for how to prepare and upload this data: https://journals.plos.org/plosbiology/s/figures#loc-blot-and-gel-reporting-requirements

d) *Blurb*

Please also provide a blurb which (if accepted) will be included in our weekly and monthly Electronic Table of Contents, sent out to readers of PLOS Biology, and may be used to promote your article in social media. The blurb should be about 30-40 words long and is subject to editorial changes. It should, without exaggeration, entice people to read your manuscript. It should not be redundant with the title and should not contain acronyms or abbreviations. For examples, view our author guidelines: https://journals.plos.org/plosbiology/s/revising-your-manuscript#loc-blurb

e) *Protocols deposition*

To enhance the reproducibility of your results, we recommend that if applicable you deposit your laboratory protocols in protocols.io, where a protocol can be assigned its own identifier (DOI) such that it can be cited independently in the future. For instructions see: https://journals.plos.org/plosbiology/s/submission-guidelines#loc-materials-and-methods

Thank you again for your submission to our journal. We hope that our editorial process has been constructive thus far, and we welcome your feedback at any time. Please don't hesitate to contact us if you have any questions or comments.

Sincerely,

Ines

--

Ines Alvarez-Garcia, PhD

Senior Editor

PLOS Biology

ialvarez-garcia@plos.org

------------------------------------

Previews Review Commons reviews

Rev. 1:

**Summary:**

In this work, Tripathi et al address the open question of how the Fat/Ds pathway affects organ shape, using the Drosophila wing as a model. The Fat/Ds pathway is a conserved but complex pathway, interacting with Hippo signalling to affect growth and providing planar cell polarity that can influence cellular dynamics during morphogenesis. Here, authors use genetic perturbations combined with quantification of larval, pupal, and adult wing shape and laser ablation to conclude that the Ft/Ds pathway affects wing shape only during larval stages in a way that is at least partially independent of its interaction with Hippo and rather due to an effect on tissue tension and myosin II distribution. Overall the work is clearly written and well presented. I only have a couple major comments on the limitations of the work.

**Major comments:**

1. Authors conclude from data in Figures 1 and 2 that the Fat/Ds pathway only affects wing shape during larval stages. When looking at the pupal wing shape analysis in Figure 2L, however, it looks there is a difference in wt over time (6h-18h, consistent with literature), but that difference in time goes away in RNAi-ds, indicating that actually there is a role for Ds in changing shape during pupal stages, although the phenotype is clearly less dramatic than that of larval stages. No statistical test was done over time (within the genotype), however, so it's hard to say. I recommend the authors test over time - whether 6h and 18h are different in wild type and in ds mutant. I think this is especially important because there is proximal overgrowth in the Fat/Ds mutants, much of which is contained in the folds during larval stages. That first fold, however, becomes the proximal part of the pupal wing after eversion and contracts during pupal stages to elongate the blade (Aiguoy 2010, Etournay 2015). Also, according to Trinidad Curr Biol 2025, there is a role for Fat/Ds pathway in pupal stages. All of that to say that it seems likely that there would be a phenotype in pupal stages. It's true it doesn't show up in the adult wing in the experiments in Fig 1, but looking at the pupal wing itself is more direct - perhaps the very proximal effect is less prominent later, as there is potential for further development after 18hr before adulthood and the most proximal parts are likely anyway excluded in the analysis.

2. I think there needs to be a mention and some discussion of the fact that the wing is not really flat. While it starts out very flat at 72h, by 96h and beyond, there is considerable curvature in the pouch that may affect measurements of different axis and cell shape. It is not actually specified in the methods, so I assume the measurements were taken using a 2D projection. Not clear whether the curvature of the pouch was taken into account, either for cell shape measurements presented in Fig 4 or for the wing pouch dimensional analysis shown in Fig 3, 6, and supplements. Do perturbations in Ft/Ds affect this curvature? Are they more or less curved in one or both axes? Such a change could affect the results and conclusions. The extent to which the fat/ds mutants fold properly is another important consideration that is not mentioned. For example, maybe the folds are deeper and contain more material in the ds/fat mutants, and that's why the pouch is a different shape? At the very least, this point about the 3D nature of the wing disc must be raised in discussion of the limitations of the study. For the cell shape analysis, you can do a correction based on the local curvature (calculated from the height map from the projection). For the measurement of A/P, D/V axes of the wing pouch, best would be to measure the geodesic distance in 3D, but this is not reasonable to suggest at this point. One can still try to estimate the pouch height/ curvature, however, both in wild type and in fat/ds mutants.

**Minor comments:**

1. The analysis of the laser ablation is not really standard - usually one looks at recoil velocity or a more complicated analysis of the equilibrium shape using a model (e.g Shivakumar and Lenne 2016, Piscitello-Gomez 2023, Dye et al 2021). One may be able to extract more information from these experiments - nevertheless, I doubt the conclusions would change, given that that there seems to be a pretty clear difference between wt and ds (OPTIONAL).

2. Figure 7G: I think you also need a statistical test between RNAi-ds and UAS-rokCA+RNAi- ds.

3. In the discussion, there is a statement: "However, as mutation or knock down of core PCP components, including pk or sple, does not affect wing shape... 59." Reference 59 is quite old and as far as I can tell shows neither images nor quantifications of the wing shape phenotype (not sure it uses "knockdown" either - unless you mean hypomorph?). A more recent publication Piscitello-Gomez et al Elife 2023 shows a very subtle but significant wing shape phenotype in core PCP mutants. It doesn't change your logic, but I would change the statement to be more accurate by saying "mutation of core PCP components has only subtle changes in adult wing shape"

**Referee cross-commenting**

Reviewer2:

Reviewer 2 makes the statement: "The distance along the AP boundary from the pouch border to DV midline is topologically comparable to the PD length of the adult wing. The distance along the DV boundary from A border to P border is topologically comparable to the AP length of the adult wing."

I disagree - the DV boundary wraps around the entire margin of the adult wing (as correctly drawn with the pink line in Fig 2A). It is not the same as the wide axis of the adult wing (perpendicular to the AP boundary). It is not trivial to map the proximal-distal axis of the larval wing to the proximal-distal axis of the adult, due to the changes in shape that occur during eversion. Thus, I find it much easier to look at the exact measurement that the authors make, and it is much more standard in the field, rather than what the reviewer suggests. Alternatively, one could I guess measure in the adult the ratio of the DV margin length (almost the circumference of the blade?) to the AP boundary length. That may be a more direct comparison.

Actually the authors leave out the term "boundary" - what they call AP is actually the AP boundary, not the AP axis, and likewise for the DV - what they measure is DV boundary, but I only noticed that in the second read-through now. Just another note, these measurements of the pouch really only correspond to the very distal part of the wing blade, as so much of the proximal blade comes from the folds in the wing disc. Therefore, a measurement of only distal wing shape would be more comparable.

Reviewer 2 states that authors cannot definitively conclude anything about mechanical tension from their reported cutting data because the authors have not looked at initial recoil velocity.

I strongly disagree. The wing disc tissue is elastic on much longer timescales than what's considered after laser ablation (even hours), and the shape of the tissue after it equilibrates from a circular cut (1-2min) can indeed be used to infer tissue stresses (see Dye et al Elife 2021, Piscitello-Gomez et al eLife 2023, Tahaei et al arXiv 2024). In the wing disc, the direction of stresses inferred from initial recoil velocity are correlated with the direction of stresses inferred from analysing the equilibrium shape after a circular cut. Rearrangements, a primary mechanism of fluidization in epithelia, does not occur within 1'. Analysing the equilibrium shape after circular ablation may be more accurate for assessing tissue stresses than initial recoil velocity - in Piscitello-Gomez et al 2023, the authors found that a prickle mutation (PCP pathway) affected initial recoil velocity but not tissue stresses in the pupal wing. Such equilibrium circular cuts have also been used to analyze stresses in the avian embryo, where it correlates with directions of stress gathered from force inference methods (Kong et al Scientific Reports 2019). The Tribolium example noted by the reviewer is on the timescale of tens to hundreds of minutes - much longer than the timescale of laser ablation retraction. It is true the analysis of the ablation presented in this paper is not at the same level as those other cited papers and could be improved. But I don't think the analysis would be improved by additional experiments doing timelapse of initial retraction velocity.

Reviewer 2 states "If cell anistropy is caused by polarized myosin activity, that activity is typically polarized along the short edges not long edges" Not true in this case. Myosin II accumulates along long boundaries (Legoff and Lecuit 2013). "Therefore, interpreting what causes the cell anistropy and how DS regulates it is difficult," Agreed - but this is well beyond the scope of this manuscript. The authors clearly show that there is a change of cell shape, at least in these two regions. Better would be to quantify it throughout the pouch and across multiple discs. Similar point for myosin quantifications - yes, polarity would be interesting and possible to look at in these data, and it would be better to do so on multiple discs, but the lack of overall myosin on the junctions shown here is not nothing. Interpreting what Ft/Ds does to influence tension and myosin and eventually tissue shape is a big question that's not answered here. I think the authors do not claim to fully understand this though, and maybe further toning down the language of the conclusions could help.

Reviewer 3:

I agree with many of the points raised by Reviewer 3, in particular that relevant for Fig 1. The additional experiments looking at myosin II localization and laser ablation in the other perturbations (Hippo and Rok mutants/RNAi) would certainly strengthen the conclusions.

Significance

I think the work provides a clear conceptual advance, arguing that the Ft/Ds pathway can influence mechanical stress independently of its interaction with Hippo and growth. Such a finding, if conserved, could be quite important for those studying morphogenesis and Fat function in this and other organisms. For this point, the genetic approach is a clear strength. Previous work in the Drosophila wing has already shown an adult wing phenotype for Ft/Ds mutations that was attributed to its role in the larval growth phase, as marked clones show aberrant growth in mutants. The novelty of this work is the dissection of the temporal progression of this phenotype and how it relates to Hippo and myosin II activation. It remains unclear exactly how Ft/Ds may affect tissue tension, except that it involves a downregulation of myosin II - the mechanism of that is not addressed here and would involve considerable more work. I think the temporal analysis of the wing pouch shape was quite revealing, providing novel information about how the phenotype evolves in time, in particular that there is already a phenotype quite early in development. As mentioned above, however, the lack of consideration of the wing disc as a 3D object is a potential limitation. While the audience is likely mostly developmental biologists working in basic research, it may also interest those studying the pathway in other contexts, including in vertebrates given its conservation and role in other processes.

Rev. 2:

The manuscript begins with very nice data from a ts sensitive period experiment. Instead of a ts mutation, the authors induced RNAi in a temperature dependent manner. The results are striking and strong. Knockdown of FT or DS during larval stages to late L3 changed shape while knockdown of FT or DS during later pupal stages did not. This indicates they are required during larval, not pupal stages of wing development for this shape effect. They did shift-up or shift-down at "early pupa stage" but precisely what stage that means was not described anywhere in the manuscript. White prepupal? Time? Likewise a shift-down was done at "late L3" but that meaning is also vague. Moreover, I was surprised to see they did not do a shift-up at the late L3 stage, to give completeness to the experiment. Why?

Looking at the "shape" of the larval wing pouch they see a difference in the mutants. The pouch can be approximated as an ellipse, but with differing topology to the adult wing. Here, they muddled the analysis. The adult wing surface is analogous to one hemisphere of the larval wing pouch, ie., either dorsal or ventral compartment. The distance along the AP boundary from the pouch border to DV midline is topologically comparable to the PD length of the adult wing. The distance along the DV boundary from A border to P border is topologically comparable to the AP length of the adult wing. They confusingly call this latter metric the "DV length" and the former metric the "AP length", and in fact they do not measure the PD length but PD+DP length. Confusing. Please change to make this consistent with earlier analysis of the adult and invert the reported ratio and divide by two. Then you would find the larval PD/AP ratio is smaller in the FT and DS mutants than wildtype, which resembles the smaller PD/AP ratio seen in the mutant adult wings. Totally consistent and also provides further evidence with the ts experiments that FT and DS exert shape effects in the larval phase of life. The remainder of the manuscript has experimental results that are more problematic, and really the authors do not figure out how the shape effect in larval stages is altered. I outline below the main problems.

1. They compare the FT DS shape phenotypes to those of mutants or knockdowns in Hippo pathway genes (Hippo is known to be downstream of FT and DS). They find these Hippo perturbations do have shape effects trending in same direction as FT and DS effects. Knockdown reduces the PD/AP ratio while overexpressing WARTS increases the PD/AP ratio. The effect magnitudes are not as strong, but then again, they are using hypomorphic alleles and RNAi, which often induces partial or hypomorphic phenotypes. The effect strength is comparable when wing pouches are young but then dissipates over time, while FT and DS effects do not dissipate over time. The complexity of the data do not negate the idea that Hippo signaling is also playing some role and could be downstream of FT and DS in all of this. But the authors really downplay the data to the point of stating "These results imply that Ds-Fat influences wing pouch shape during wing disc growth separately from its effects on Hippo signaling." I think a more expansive perspective is needed given the caveats of the experiments. Puzzlingly, this lack of taking seriously a set of complex results does not transfer to another set of experiments in which they inhibit or activate ROK, the rho kinase. When ROK is perturbed, they also see weak effects on shape when compared to FT or DS perturbation. This weakness is seen in adults, larvae, clones and in epistasis experiments. The epistasis experiment in particular convincingly shows that constitutuve ROK activation is not epistatic to loss of DS; in fact if anything the DS phenotype suppresses the ROK phenotype. These results also show that one cannot simply explain what FT and DS are doing with some single pathway or effector molecule like ROK. It is more complex than that. What I really think was needed were experiments combining FT and DS knockdown with other mutants or knockdowns in the Hippo and Rho pathways, and even combining Hippo and Rho pathway mutants with FT or DS intact, to see if there are genetic interactions (additive, synergistic, epistatic) that could untangle the phenotypic complexity.

2. Laser cutting experiments were done to see if there is anisotropy in tissue tension within the wing pouch. This was to test a favored idea that FT and DS activity generates anisotropy in tissue tension, thereby controlling overall anisotropic shape of the pouch. However there is a fundamental flaw to their laser cutting analysis. Laser cutting is a technique used to measure mechanical tension, with initial recoil velocity directly proportional to the tissue's tension. By cutting a small line and observing how quickly the edges of the cut snap apart, people can quantify the initial recoil velocity and infer the stored mechanical stress in the tissue at the time of ablation. Live imaging with high-speed microscopy is required to capture the immediate response of the tissue to the cut since initial recoil velocity occurs in the first few seconds. A kymograph is created by plotting the movement of the tissue edges over this time scale, perpendicular to the cut. The initial recoil velocity is the slope of the kymograph at time zero, representing how fast the severed edges move apart. A higher recoil velocity indicates higher mechanical tension in the tissue. However, the authors did not measure this initial recoil velocity but instead measured the distance between the severed edges at one time point: 60 seconds after cutting. This is much later than the time point at which the recoil usually begins to dissipate or decay. This decay phase typically lasts a minute or two, during which time the edges continue to separate but at a progressively slower rate. This time- dependent decay of the recoil reveals whether the tissue behaves more like a viscous fluid or an elastic solid. Therefore, the distance metric at 60 seconds is a measurement of both tension and the material properties of the cells. One cannot know then whether a difference in the distance is due to a difference in tension or fluidity of the cells. If the authors made measurements of edge separation at several time points in the first 10 seconds after ablation, they can deconvolute the two. Otherwise their analysis is inconclusive. Anisotropy in recoil could be caused by greater tissue fluidity along one axis. Observing a gradient of cell fluidity in a tissue along one axis of a tissue has been observed in the amnioserosa of Tribolium for example. (Related and important point - was the anisotropy of recoil oriented along the PD or AP axis or not oriented to either axis, this key point was never stated). The authors cannot definitively conclude anything about mechanical tension from their reported cutting data.

3. They measured the eccentricity of wing pouch cells near the pouch border, and found they were highly anisotropic compared to DS mutant cells at comparable locations. Cells were elongated but again what if either axis (PD or AP) they were elongated along was never stated. If cell anistropy is caused by polarized myosin activity, that activity is typically polarized along the short edges not long edges. Thus, recoil velocity after laaser cutting would be stonger along the axis aligned with short cell edges. It looks like the cutting anisotropy they see is greater along the axis aligned with long cell edges. Of course, if the cell anisotropy is caused by a pulling force exerted by the pouch boundary, then it would stretch the cells. This would in fact fit their cutting data. But then again, the observed cell anisotropy could also be caused by variation in the fluid-solid properties of the wing cells as discussed earlier. Compression of the cells then would deform them anisotropically and produce the anisotropic shapes that were observed, Therefore, interpreting what causes the cell anistropy and how DS regulates it is difficult,

4. The imaging and analysis of the myosin RLC by GFP tagging is also flawed. SQH-GFP is a tried and true proxy for myosin activity in Drosophila. Although the authors image the wing pouch of wildtype and DS mutants. they did so under low magnification to image the entire pouch. This gives a "low-res" perspective of overall myosin but what they needed to do was image at high magnification in that proximal region of the pouch and see if Sqh-GFP is polarized in wildtype cells along certain cell edges aligned with an axis. And if such a polarity is observed, is it present or absent in the DS mutant. From the data shown in Figure 5, I cannot see any significant difference between wildtype and knocked down samples at this low resolution. Any difference, if there is any, is not really interpretable.

In conclusion, the manuscript has multiple problems that make it imposiible for the authors to make the claims they make in the current manuscript. And even if they calibrated their interpretations to fit the data, there is not much of a simple clear picture as to how FT and DS regulate pouch eccentricity in the larval wing.

Significance

This manuscript describes experiments studying the role that the protocadherins FAT and DACHSOUS play in determining the two dimensional "shape" of the fruit fly wing. By "shape", the manuscript really means how much the wing's outline, when approximated as an ellipse, deviates from a circle. The elliptical approximations of FT and DS mutant wings more closely resemble a circle compared to the more eccentric wildtype wings. This suggests the molecules contribute to anisotropic growth in some way. A great deal of attention has been paid on how FT and DS regulate overall organ growth and planar cell polarity, and the Irvine lab has made extensive contributions to these questions over the years. Somewhat understudied is how FT and DS regulate wing shape, and this manuscript focuses on that. It follows up on an interesting result that the Irvine lab published in 2019, in which mud mutants randomized spindle pole orientation in wing cells but did not change the eccentricity of wings, ruling out biased cell division orientation as a mechanism for the anisotropic growth.

Rev. 3:

**Summary**

The authors investigate the mechanisms underlying epithelial morphogenesis using the Drosophila wing as a model system. Specifically, they analyze the contribution of the conserved Fat/Ds pathway to wing shape regulation. The main claim of the manuscript is that Ds/Fat controls wing shape by regulating tissue mechanical stress through MyoII levels, independently of Hippo signaling and tissue growth.

**Major Comments**

To support their main conclusions, the authors should address the following major points and consider additional experiments where indicated. Most of the suggested experiments are feasible within a reasonable timeframe, while a few are more technically demanding but would substantially strengthen the manuscript's central claims.

*Figure 1:*

The authors use temperature-sensitive inactivation of Fat or Ds to determine the developmental window during which these proteins regulate wing shape. To support this claim, it is essential to demonstrate that upon downshift during early pupal stages, Ds or Fat protein levels are restored to normal. For consistency, please include statistical analyses in Figure 1P and ensure that all y-axis values in shape quantifications start at 1.

*Figure 2:*

The authors propose that wing shape is regulated by Fat/Ds during larval development. However, Figure 2L suggests that wing elongation occurs in control conditions between 6 and 12 h APF, while this elongation is not observed upon Ds RNAi. The authors should therefore perform downshift experiments while monitoring wing shape during the pupal stage to substantiate their main claim. In addition, equivalent data for Fat loss of function should be included to support the assertion that Fat and Ds act similarly.

*Figure 3:*

The authors state that "These observations indicate that Ds-Fat signaling influences wing shape during the initial formation of the wing pouch, in addition to its effects during wing growth." This conclusion is not fully supported, as the authors only examine wing shape at 72 h AEL. At this stage, fat or ds mutant wings already display altered morphology. The authors could only make this claim if earlier time points were fully analyzed. In fact, the current data rather suggest that Ds function is required before 72 h AEL, as a rescue of wing shape is observed between 72 and 120 h AEL.

*Figure 4:*

The authors state that "The influence of Ds-Fat on wing shape is not explained by Hippo signaling." However, this conclusion is not supported by their data, which show that partial loss of ex or hippo causes clear defects in wing shape. In addition, the initial wing shape is affected in wts and ex mutants, and hypomorphic alleles were used for these experiments. Therefore, the main conclusion requires revision. It would be useful to include a complete dataset for hippo RNAi, ex, and wts conditions in Figure S1. The purpose and interpretation of the InR^CA experiments are also unclear. While InR^CA expression can increase tissue growth, Hippo signaling has functions beyond growth control. Whether Hippo regulates tissue shape through InR^CA-dependent mechanisms remains to be clarified.

*Figure 5:*

This figure presents images of MyoII distribution, but no quantification across multiple samples is provided. Moreover, the relationship between changes in tissue stress and MyoII levels remains unclear. Performing laser ablation and MyoII quantification on the same samples would provide stronger support for the proposed conclusions.

*Figure 6:*

It is unclear when Rok RNAi and Rok^CA misexpression were induced. To substantiate their claims, the authors should measure both MyoII levels and mechanical tension under the different experimental conditions in which wing shape was modified through Rok modulation (i.e. the condition shown in Fig. 7G). For comparison, fat and ds data should be added to Fig 6H. Overall, the effects of Rok modulation appear milder than those of Fat manipulation. Given that Dachs has been shown to regulate tension downstream of Fat/Ds, it would be informative to determine whether tissue tension is altered in dachs mutant wings and to assess the relative contribution of Dachs- versus MyoII-mediated tension to wing shape control. It would also be interesting to test whether Rok activation can rescue dachs loss-of- function phenotypes.

*Figure 7:*

The authors use genetic interactions to support their claim that Fat controls wing shape independently of Hippo signaling. However, these interactions do not formally exclude a role for Hippo. Moreover, previous work has shown that tissue tension regulates Hippo pathway activity, implying that any manipulation of tension could indirectly affect Hippo and growth. To provide more direct evidence, the authors should further analyze MyoII localization and tissue tension under the various experimental conditions tested (as also suggested above).

Significance

How organ growth and shape are controlled remains a fundamental question in developmental biology, with major implications for our understanding of disease mechanisms. The Drosophila wing has long served as a powerful and informative model to study tissue growth and morphogenesis. Work in this system has been instrumental in delineating the conserved molecular and mechanical processes that coordinate epithelial dynamics during development. The molecular regulators investigated by the authors are highly conserved, suggesting that the findings reported here are likely to be of broad biological relevance.

Previous studies have proposed that anisotropic tissue growth regulates wing shape during larval development and that such anisotropy induces mechanical responses that promote MyoII localization (Legoff et al., 2013, PMID: 24046320; Mao et al., 2013, PMID: 24022370). The Ds/Fat system has also been shown to regulate tissue tension through the Dachs myosin, a known modulator of the Hippo/YAP signaling pathway. As correctly emphasized by the authors, the respective contributions of anisotropic growth and mechanical tension to wing shape control remain only partially understood. The current study aims to clarify this issue by analyzing the role of Fat/Ds in controlling MyoII localization and, consequently, wing shape. This represents a potentially valuable contribution. However, the proposed mechanistic link between Fat/Ds and MyoII localization remains insufficiently explored. Moreover, the role of MyoII is not fully discussed in the broader context of Dachs function and its known interactions with MyoII (Mao et al., 2011, PMID: 21245166; Bosveld et al., 2012, PMID: 22499807; Trinidad et al., 2024, PMID: 39708794). Most importantly, the experimental evidence supporting the authors' conclusions would benefit from further strengthening. It should also be noted that disentangling the relative contributions of anisotropic growth and MyoII polarization to tissue shape and size remains challenging, as MyoII levels are known to increase in response to anisotropic growth (Legoff et al., 2013; Mao et al., 2013), and mechanical tension itself can modulate Hippo/YAP signaling (Rauskolb et al., 2014, PMID: 24995985).

# Author comment: Author response to Decision Letter 2

**DOI:** 10.1371/journal.pbio.3003883.r005

22 Apr 2026

Attachment Submitted filename: Response to Reviews_v6.docx

# Peer review: Decision Letter 2

**DOI:** 10.1371/journal.pbio.3003883.r006

**Authors:** Ines Alvarez-Garcia, Ines Alvarez-Garcia, Ines Alvarez-Garcia

5 Jun 2026

Dear Dr Irvine,

Thank you for your patience while we considered your revised manuscript entitled "Dachsous-Fat Signaling Shapes the Drosophila Wing through Mechanical Forces" for publication as a Research Article at PLOS Biology. This revised version of your manuscript has been evaluated by the PLOS Biology editors, the Academic Editor and one of the original reviewers.

Based on the review (attached below), we are likely to accept this manuscript for publication, provided you address the minor point raised by Reviewer 1 (point 2) and the data and other policy-related requests stated below my signature.

As you address these items, please take this last chance to review your reference list to ensure that it is complete and correct. If you have cited papers that have been retracted, please include the rationale for doing so in the manuscript text, or remove these references and replace them with relevant current references. Any changes to the reference list should be mentioned in the cover letter that accompanies your revised manuscript.

In addition to these revisions, you may need to complete some formatting changes, which you will receive in a follow up email. A member of our team will be in touch with a set of requests shortly. If you do not receive a separate email within a few days, please assume that checks have been completed, and no additional changes are required.

We expect to receive your revised manuscript within two weeks.

To submit your revision, please go to https://www.editorialmanager.com/pbiology/ and log in as an Author. Click the link labelled 'Submissions Needing Revision' to find your submission record. Your revised submission must include the following:

- a cover letter that should detail your responses to any editorial requests, if applicable, and whether changes have been made to the reference list

- a Response to Reviewers file that provides a detailed response to the reviewers' comments (if applicable, if not applicable please do not delete your existing 'Response to Reviewers' file.)

- a track-changes file indicating any changes that you have made to the manuscript.

NOTE: If Supporting Information files are included with your article, note that these are not copyedited and will be published as they are submitted. Please ensure that these files are legible and of high quality (at least 300 dpi) in an easily accessible file format. For this reason, please be aware that any references listed in an SI file will not be indexed. For more information, see our Supporting Information guidelines:

https://journals.plos.org/plosbiology/s/supporting-information

*Published Peer Review History*

Please note that you may have the opportunity to make the peer review history publicly available. The record will include editor decision letters (with reviews) and your responses to reviewer comments. If eligible, we will contact you to opt in or out. Please see here for more details:

https://plos.org/published-peer-review-history/

*Press*

Should you, your institution's press office or the journal office choose to press release your paper, please ensure you have opted out of Early Article Posting on the submission form. We ask that you notify us as soon as possible if you or your institution is planning to press release the article.

*Protocols deposition*

To enhance the reproducibility of your results, we recommend that if applicable you deposit your laboratory protocols in protocols.io, where a protocol can be assigned its own identifier (DOI) such that it can be cited independently in the future. Additionally, PLOS ONE offers an option for publishing peer-reviewed Lab Protocol articles, which describe protocols hosted on protocols.io. Read more information on sharing protocols at https://plos.org/protocols?utm_medium=editorial-email&utm_source=authorletters&utm_campaign=protocols

Please do not hesitate to contact me should you have any questions.

Sincerely,

Ines

--

Ines Alvarez-Garcia, PhD

Senior Editor

PLOS Biology

ialvarez-garcia@plos.org

------------------------------------------------------------------------

DATA POLICY:

You may be aware of the PLOS Data Policy, which requires that all data be made available without restriction: http://journals.plos.org/plosbiology/s/data-availability. For more information, please also see this editorial: http://dx.doi.org/10.1371/journal.pbio.1001797

Note that we do not require all raw data. Rather, we ask that all individual quantitative observations that underlie the data summarized in the figures and results of your paper be made available in one of the following forms:

1) Supplementary files (e.g., excel). Please ensure that all data files are uploaded as 'Supporting Information' and are invariably referred to (in the manuscript, figure legends, and the Description field when uploading your files) using the following format verbatim: S1 Data, S2 Data, etc. Multiple panels of a single or even several figures can be included as multiple sheets in one excel file that is saved using exactly the following convention: S1_Data.xlsx (using an underscore).

2) Deposition in a publicly available repository. Please also provide the accession code or a reviewer link so that we may view your data before publication.

Regardless of the method selected, please ensure that you provide the individual numerical values that underlie the summary data displayed in the following figure panels as they are essential for readers to assess your analysis and to reproduce it:

Fig. 1D, S; Fig. 2E, O; Fig. 3D; Fig. 4F-I; Fig. 5F, I’, J’; Fig. 6D, H, L; Fig. 7G, L, M; Fig. 8G, K-N; Fig. S2E; Fig. S3I-L; Fig. S4D, E, G; Fig. S5E, H, I; Fig. S6A-D, J and Fig, S7F, G, J, K

NOTE: the numerical data provided should include all replicates AND the way in which the plotted mean and errors were derived (it should not present only the mean/average values).

Please also ensure that figure legends in your manuscript include information on WHERE THE UNDERLYING DATA CAN BE FOUND, and ensure your supplemental data file/s has a legend.

Please ensure that your Data Statement in the submission system accurately describes where your data can be found.

------------------------------------------------------------------------

Reviewers' comments

Rev. 1:

Overall, the revision is responsive to my prior comments. The most critical methodological concern is convincingly resolved by the addition of recoil velocity data that corroborates the circular ablation results. The myosin polarity analysis and the expanded Dachs experiments are genuine advances.

1. I stated the timing of temperature shifts was vague and a late L3 shift-up was missing.

The authors added precise timing definitions and the requested late L3 shift-up experiment. Figure 1R adds the missing shift-up.

2. I argued the shape metric was topologically mislabeled and should be inverted.

The manuscript now clearly states in the Figure 2 legend and main text that the DV length corresponds to the wing circumference and the AP length is proportional to twice the wing length. This is a defensible clarification. However, the authors declined to invert the ratio, which will leave some readers confused since a larger DV/AP ratio in their system corresponds to a rounder wing, the opposite of intuition. Either invert the ratio as I had previously requested or add text explicitly flagging this counter-intuitive metric.

3. I was concerned that the authors overstated that Hippo is irrelevant. The data show Hippo does contribute.

The authors revised the conclusion to "Hippo signaling contributes to, but does not fully explain, the influence of ds or fat on adult wing shape." They added wing size measurements (new Fig S3) and clarified allele strengths. The revised language is appropriately hedged. The addition of wts RNAi adult wings and hpo/wts RNAi wing discs in the new Fig S3 strengthens the ms. The key mechanistic distinction — that Hippo knockdown and Ds-Fat knockdown have opposite effects on myosin distribution (Fig S7) is a compelling new addition that supports their conclusion.

4. I was concerned that the 60 second distance measurement conflates tension with cell fluidity. Initial recoil velocity should have been measured.

The authors added recoil velocity measurements for wildtype and ds mutants (new Fig S6), showing consistency with circular ablation results. The new Fig S6 shows recoil velocity data confirming that proximal junctions have lower tension, and D-V boundary junctions have higher tension in ds mutants, corroborating the circular ablation inference. The axis of anisotropy (proximal vs D-V boundary) is now also explicitly stated throughout.

5. I pointed out that the axis along which cells were elongated was never specified.

The authors revised text and figures to clarify orientation of regions shown. The manuscript now clearly distinguishes proximal regions from D-V boundary regions, and the schematic in Figure 4A explicitly maps these locations on the wing disc.

6. I was concerned that low-magnification Sqh:GFP images were uninterpretable. High-resolution polarization analysis was needed.

The authors added high-resolution myosin images and quantitative assessments of myosin junctional polarity in control and ds mutants (revised Fig 5, including new panels I and J with rose plots). The polarity analysis showing loss of radial vs tangential myosin bias in ds mutants is a meaningful new result that goes beyond what was requested.

# Author comment: Author response to Decision Letter 3

**DOI:** 10.1371/journal.pbio.3003883.r007

12 Jun 2026

Attachment Submitted filename: Revised Response to Reviews_v1.docx

# Editor report: Decision Letter 3

**DOI:** 10.1371/journal.pbio.3003883.r008

**Authors:** Ines Alvarez-Garcia, Ines Alvarez-Garcia, Ines Alvarez-Garcia, Ines Alvarez-Garcia

17 Jun 2026

Dear Dr Irvine,

Thank you for the submission of your revised Research Article entitled "Dachsous-Fat Signaling Shapes the Drosophila Wing through Mechanical Forces" for publication in PLOS Biology. On behalf of my colleagues and the Academic Editor, Nic Tapon, I am delighted to let you know that we can in principle accept your manuscript for publication, provided you address any remaining formatting and reporting issues. These will be detailed in an email you should receive within 2-3 business days from our colleagues in the journal operations team; no action is required from you until then. Please note that we will not be able to formally accept your manuscript and schedule it for publication until you have completed any requested changes.

Please take a minute to log into Editorial Manager at http://www.editorialmanager.com/pbiology/, click the "Update My Information" link at the top of the page, and update your user information to ensure an efficient production process.

PRESS

We frequently collaborate with press offices. If your institution or institutions have a press office, please notify them about your upcoming paper at this point, to enable them to help maximise its impact. If the press office is planning to promote your findings, we would be grateful if they could coordinate with biologypress@plos.org. If you have previously opted in to the early version process, we ask that you notify us immediately of any press plans so that we may opt out on your behalf.

We also ask that you take this opportunity to read our Embargo Policy regarding the discussion, promotion and media coverage of work that is yet to be published by PLOS. As your manuscript is not yet published, it is bound by the conditions of our Embargo Policy. Please be aware that this policy is in place both to ensure that any press coverage of your article is fully substantiated and to provide a direct link between such coverage and the published work. For full details of our Embargo Policy, please visit http://www.plos.org/about/media-inquiries/embargo-policy/.

Many congratulations and thanks again for choosing PLOS Biology for publication and supporting Open Access publishing. We look forward to publishing your study.

Sincerely,

Ines

--

Ines Alvarez-Garcia, PhD

Senior Editor

PLOS Biology

ialvarez-garcia@plos.org
